# A spatial perspective on the impact of official development assistance on sustainable development goals

**DOI:** 10.1038/s41598-026-35544-z

**Published:** 2026-02-01

**Authors:** Shuang Liu, Tim Ölkers, Oliver Mußhoff, Xiaohua Yu

**Affiliations:** 1 School of Agricultural Economics and Rural Development, Renmin University of China, Beijing, China; 2 Department of Agricultural Economics, Humboldt University, Berlin, Germany; 3 Department of Agricultural Economics and Rural Development, University of Göttingen, Göttingen, Germany

**Keywords:** Sustainable development goals, Official development assistance, Spatial autocorrelation, Aid effectiveness, Development studies, Environmental social sciences, Environmental studies, Geography, Geography

## Abstract

The United Nations (UN)’s 2030 Agenda for 17 Global Sustainable Development Goals (SDGs) is a critical framework for advancing environmental sustainability and human development. Official Development Assistance (ODA) serves as a key source of financing for the SDGs, particularly in developing countries. This study investigates the impact of ODA on various aspects of SDG performance, emphasizing the need for a holistic approach that accounts for the diverse needs, uneven progress, and spatial interdependencies among UN member states between 2000 and 2021. Our findings show that ODA statistically significantly supports basic goals such as SDG 1 (no poverty) and SDG 2 (zero hunger), but has limited, or in some cases negative, effects on other goals, including SDG 8 (decent work), SDG 9 (industry and innovation), SDG 15 (life on land), and SDG 16 (peace and institutions). The results also reveal substantial heterogeneity across country groups, underscoring the need for ODA to broaden its focus to underfunded areas and adopt more context-specific strategies that recognize spatial dynamics, manage trade-offs, and prioritize SDG pathways aligned with country-specific capacities and priorities.

## Introduction

In 2015, United Nation (UN) member states adopted the 2030 Agenda for Sustainable Development, which outlines a comprehensive global plan of action for “people, planet, and prosperity”. This plan comprises of 17 global Sustainable Development Goals (SDGs) and 169 targets to be achieved by 2030. These goals encompass economic, environmental, and social dimensions^1^ and are considered to be the most crucial factors in understanding and achieving environmental and human development^2^.

The focus of quantitative assessment for SDGs has largely been on setting targets, indicators, and metrics for monitoring overall success^3–8^. Several methodological studies have been conducted on indicators used to measure SDGs and monitor progress, but they all conclude that further research in relation to SDGs is needed^9–15^. Specifically, the progress of the SDGs for UN member states is assessed in the Sustainable Development Report 2022 (hereafter, SDR2022)^7^, which remains the most recent and comprehensive global assessment of SDG advancement. The SDR2022 provides detailed, country-level evaluations for all 17 SDGs and compiles these into an aggregate SDG index, enabling robust cross-country comparisons and systematic monitoring of progress toward the 2030 Agenda.

Research on the SDGs spans a wide range of disciplines^16^, including global welfare^3^, climate change^17,18^, ecological footprints and international spillovers^8^, and pathways for achieving specific goals^19–22^. A growing body of work also examines trade-offs and complementarities among the SDGs. For example, SDG 1 (no poverty) and SDG 2 (zero hunger) tend to lie downstream, benefiting from progress in other areas, whereas SDG 12 (responsible consumption and production) and SDG 17 (partnerships) function as upstream enablers of broader progress^23^. Other studies similarly highlight the coexistence of synergies and trade-offs across goals^3,24^. A parallel strand of literature underscores that achieving many SDGs requires substantial external financing, particularly through foreign direct investment (FDI) and official development assistance (ODA)^25–27^. In countries such as the Central African Republic, Somalia, and Yemen, ODA amounts to more than one-third of gross national income^28^, illustrating its critical role in supporting public budgets and SDG-related initiatives. Despite this importance, the effectiveness of ODA in advancing sustainable development remains contested, leaving open the central question of how ODA contributes to progress across the SDGs.

A number of studies have demonstrated the positive impact of ODA on the SDGs. For instance, one study finds that aid for agricultural education, research, and services, as well as agricultural water, effectively reduces child stunting (SDG 2 (zero hunger) and SDG 3 (good health and well-being))^29^. Additionally, higher per capita aid to education leads to a statistically significant increase in primary school enrollment (SDG 4 (quality education))^30^. Chinese aid decreases infant mortality at the country level but increases infant mortality at the sub-national level compared to the country average (SDG 3 (good health and well-being))^31^. High gender gaps in education and health are associated with higher aid allocation in these sectors and aid overall^32^. Donors appear to be more responsive to inequalities in countries that provide women with good legal rights, leading to greater political representation of women and higher aid flows (SDG 3 (good health and well-being), SDG 4 (quality education), SDG 5 (gender equality), and SDG 10 (reduced inequalities)). These sector-specific differences underscore the importance of considering the nature and scope of interventions when assessing the impact of ODA on development outcomes, including progress toward specific SDGs.

However, the overall effectiveness of ODA in promoting broad-based sustainable development remains contested, and several studies point to limited or even negative impacts in some areas. In fact, cross-country evidence has long identified an “aid paradox”. Despite decades of large aid inflows, many recipient countries have seen little improvement in composite development outcomes. For example, aid has not led to sustained economic growth in Malawi^33^, a finding with implications for SDG 8 (decent work and economic growth). Likewise, taking a long-run perspective, ODA generally has a statistically insignificant or even slightly negative effect on per capita income, particularly in highly aid-dependent countries^34^. One reason is that ODA can inadvertently undermine domestic investment and productivity: aid may crowd out domestic savings and only boost consumption, yielding minimal sustainable growth^35^. In addition, large aid inflows can induce currency overvaluation (a phenomenon akin to Dutch Disease), which harms export competitiveness and industrial development. One study, for instance, argues that aid-driven real exchange rate appreciation has negative effects on manufacturing growth, impeding progress on SDG 9 (industry, innovation and infrastructure)^36^. These findings suggest that more ODA does not automatically translate into higher overall SDG index scores or economic prosperity; on the contrary, without the right conditions, aid can falter in stimulating inclusive growth.

ODA’s impact on environmental sustainability, SDG 15 (life on land), has also been mixed, with some troubling evidence of unintended consequences. While aid for environmental and conservation projects is intended to protect ecosystems, its effectiveness depends greatly on context. One study examines conservation aid across 42 African countries and find that such aid was associated with higher rates of deforestation in the short term (one to two years later)^37^. They suggest this paradoxical result may stem from aid-induced displacement of resource exploitation (e.g. stricter protections in one area pushing deforestation to another) or other implementation challenges, especially where governance is weak. Furthermore, ODA can influence governance, peace, and institutional quality, the focus of SDG 16 (peace, justice and strong institutions), in adverse ways. Large volumes of aid, particularly in countries with poor governance, risk creating dependency and rent-seeking, thereby weakening institutional development^35^. For example, another study finds that high aid dependence in sub-Saharan Africa (SSA) worsened the quality of governance, as massive aid inflows can erode accountability and bureaucratic effectiveness^38^. One mechanism is through corruption: some studies document that aid can increase corruption levels in recipient countries (e.g. via capture of funds or patronage networks)^38^, which directly undermines the rule of law and trust in public institutions. In the most extreme cases, aid may even aggravate conflict dynamics. One study provides evidence that surges in U.S. food aid statistically significantly prolonged civil conflicts in recipient countries, as warring factions diverted aid supplies to sustain their efforts^39^. Such findings illustrate that humanitarian assistance, despite good intentions, can inadvertently fuel violence and instability, clearly at odds with the aims of SDG 16.

One study argues that the political influence on aid allocation can reduce aid effectiveness when the recipient country has a weak macroeconomic position^40^. The study highlights the importance of ensuring that aid is allocated based on needs rather than political considerations. Meanwhile, another study examines the extent to which climate-related ODA aligns with the implementation of the SDGs and with recipient countries’ climate-related priorities^41^ . The study finds that while climate-related ODA contributes to multiple SDGs, there is still room for improvement in the alignment between donors’ and recipients’ priorities. This suggests the need for better coordination between donors and recipients to ensure that aid is allocated in line with recipient countries’ priorities. One study examines the relationship between the Millennium Development Goals (MDGs) and aid targeting and finds a nuanced picture. While some MDGs, such as combating HIV/AIDS, have shaped resource allocation, other MDGs, such as primary education, have a gap in aid allocation^42^.

These mentioned studies form an important cornerstone for our analysis of the impact of ODA on SDGs to support more informed decision-making. Although ODA has the potential to drive sustainable development, existing studies suggest that a more comprehensive analysis and evaluation of ODA’s effectiveness towards achieving SDGs is necessary. Thus, the purpose of this paper is to emphasize the importance of a holistic approach to ODA in general and aid allocation in particular that considers the diverse needs and heterogeneous performance of SDGs as tracked by the SDR2022.

Moreover, previous studies recognize the impact of international spillover effects on achieving the SDGs. The authors of the SDR2022 also discuss the concept of international spillover effects and raise the question of whether the SDGs and their fulfillment have a spatial spillover effect that can be quantified econometrically^7^. These spillovers, also referred to as positive or negative externalities, might occur when the actions of one country or region affect the attainment of the SDGs in neighboring countries or regions. For instance, if a neighboring country experiences environmental degradation, this may have negative consequences for attaining SDGs related to climate action or clean water and sanitation. One body of literature argues that geographic conditions such as access to rivers, the prevalence of diseases, and the productivity of rain-fed agriculture influence the economic development of a country^43^. One study shows that geography statistically significant affects economic growth using a comprehensive sample of African countries^44^. Notably, rugged terrain hinders trade and other productive activities, which harms economic growth. Conversely, the rough terrain in some parts of Africa protected inhabitants who were raided during the slave trade. Such findings suggest that similar geographical characteristics might lead to similar patterns of SDG fulfillment. Consequently, questions have emerged regarding the extent to which neighboring countries’ SDG performance affects each other and to what degree a country’s SDG performance is influenced by its neighbors. Gaining a comprehensive understanding of the similarities and potential spatial autocorrelation between countries’ achievements of SDGs is crucial for developing and implementing precise, target group-oriented policy measures.

In response, the aim of this paper is to address two main research questions. Firstly, the paper investigates the extent to which the fulfillment of the SDGs exhibits spatial autocorrelation. To answer this question, the paper employs a nearest neighbor weighting matrix and calculates the Moran’s I coefficient to estimate the correlation between spatial proximity and SDG fulfillment. This analysis provides insights into the potential spatial spillover effects of SDG fulfillment. Secondly, the paper aims to estimate the impact of net ODA received on SDG performance, measured by SDG indices from the SDR2022, using a more sophisticated methodology that accounts for spatial autocorrelation and endogeneity. To achieve the goal of the second question, we have utilized panel data to track the changing trend of both the SDG indices and net ODA received. If spatial autocorrelations do exist in the achievement of SDGs globally, then the conventional ordinary least squares regression (OLS) may not accurately estimate the net impact of ODA on the SDGs. As such, a spatial econometric model that accounts for these spillovers is more appropriate. This is achieved by using a spatial autocorrelation (SAC) model. To ensure accurate estimates of the net impact of ODA on the SDGs, this paper controls for other relevant factors, including income level and governance. Additionally, the analysis incorporates individual and time fixed effects to account for unobserved factors. To address potential endogeneity, an instrumental variable (IV) approach is used, with lagged ODA serving as the instrument^45^. Other alternative weighting matrix and spatial regression models are dicusssed and conducted to ensure the robustness.

To the best of our knowledge, this paper is the first to investigate the spatial autocorrelation of SDG fulfillment, employing a nearest neighbor weighting matrix and Moran’s I coefficient to estimate the correlation between spatial proximity and SDG performance. Furthermore, this study is the first comprehensive analysis of the impact of net ODA received on the 17 SDG indices and on one overall SDG index using panel data and SAC models. Moreover, this study accounts for the spillover effects in SDG fulfillment using a comprehensive dataset. The estimation strategy employed in this paper contributes to the methodological literature by combining theoretical considerations with econometric elements. By adopting a comprehensive approach, this study provides policymakers and practitioners with accurate and relevant information to enhance their decision-making. The results of this study could provide insights into how ODA can be used more effectively to achieve the SDGs and promote sustainable development.

The rest of the paper is structured as follows. Section 2 presents the methodology for measuring the spatial autocorrelation of global SDGs achievement and for measuring the impact of ODA on the 17 SDG indices and on one overall SDG index. Section 3 describes the data sources. Subsection 4.1 presents the changing trend of the SDG indices and the summary statistics for control variables. Subsection 4.2 discuss the spatial correlation of SDGs, while Subsection 4.3 presents the results of the analysis of the panel data over the period 2000 to 2021. Subsection 4.4 presents the heterogeneity in the results. Finally, Section 5 discusses the results and its implications of the results for policy, while in Section 6 we provide concluding remarks.

## Methodology

### Measuring spatial correlation

A body of literature highlights that the geographical situation might affect, for instance, the economic development of a country^43,44^. “Geography matters because it affects the profitability of various kinds of economic activities, including agriculture, mining, and industry; the health of the population; and the desirability of living and investing in particular place”^43^.

There are two types of Moran’s I statistics: global and local. The global Moran’s I is a measure describing the overall spatial relationship across all geographic units for the entire study area while the local Moran’s I provides information about the specific locations and patterns of spatial autocorrelation within the study area. Based on this body of literature, this paper uses global Moran’s I statistics as indexes of spatial autocorrelation to test the spatial autocorrelation for SDGs score. The global Moran’s I can be calculated from the following formula^46^:

1 $$\begin{aligned} I=\frac{n}{\sum _{i=1}^n \sum _{j=1}^n w_{i j}} \frac{\sum _{i=1}^n \sum _{j=1}^n w_{i j}\left( y_i-{\bar{y}}\right) \left( y_j-{\bar{y}}\right) }{\sum _{i=1}^n\left( y_i-{\bar{y}}\right) ^2} \end{aligned}$$

where *n* is the number of spatial units. ${\overline{y}}$ is the mean value of the SDGs score across the entire data. $y_i$ and $y_j$ are the value of the SDGs score for country *i* and *j*. $w_{i j}$ is the weight matrix which describes the relationship for each pair of country *i* and *j*.

The values of global Moran’s I range from “-1” to “1”. If Moran’s I is above 0, there is positive spatial correlation between SDGs score; the larger this value is, the stronger the correlation will be. Inversely, if Moran’s I is below 0, negative spatial correlation exists. The statistical significance of Moran’s I can be tested using permutation tests. In the paper, statistical inference is based on 99,999 permutations^47^.

**Fig. 1:**
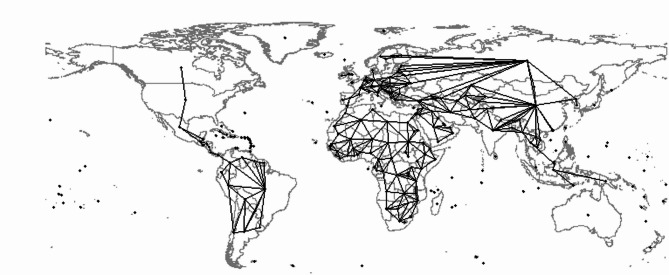
The map of geographical contiguities. Note: The global administrative areas were obtained from the Food and Agriculture Organization of the United Nations^49^. Source: Own visualization based on the software R, Version: 2022.02.0

Constructing a weight matrix is the first step for calculating Moran’s I and conducting spatial analysis. In this study, we constructed the weight matrix based on geographical contiguities, which defines neighbors by the presence of a common edge between two spatial units^48^. Figure 1 displays the geographical contiguities map used in this paper. The global administrative areas were obtained from the Food and Agriculture Organization of the United Nations^49^. The administrative boundaries at the level 1 data set are part of the Global Administrative Areas (GADM) 3.6 vector data set series which includes distinct data sets representing administrative boundaries for all countries (regions) in the world. We utilized R to calculate a sophisticated geographical weighting matrix based on the contiguities map. Specifically, we derived a basic binary geographical weighting matrix where each value in the matrix equals 1 if the two countries share a common border and 0 otherwise. This matrix allowed us to examine the interrelationships and dependencies among countries, taking their geographic proximity and potential influences on each other into account.

Global Moran’s I is used as the primary measure of spatial autocorrelation because it provides a well-established and parsimonious global statistic for detecting overall spatial dependence across countries^46^. This choice aligns with our objective of examining broad cross-country spatial patterns in SDG performance rather than identifying local clusters. Although alternative indicators such as Local Indicators of Spatial Association (LISA)^50^ can capture local heterogeneity, our analytical focus is on global spillover effects. Acknowledging that Moran’s I is sensitive to the specification of the spatial weight matrix, we further constructed several alternative weight matrices, including distance-based weights and k-nearest-neighbor weights, to assess the robustness of our chosen contiguity matrix. These alternative matrices are widely used in spatial econometric applications and capture different forms of spatial proximity. As shown in Table A5 in the Supplementary Material, the contiguity-based matrix produces the highest Moran’s I values across the SDG indices, indicating that it best represents the relevant spatial dependence structure in our cross-country setting.

### Spatial autocorrelation models

The SAC models were first introduced in the context of a linear spatial model, which includes exogenous variables, a spatially lagged dependent variable, and a spatially lagged error term^51^. In this study, we utilize the SAC model with individual fixed effects on the country level and time fixed effects, which allows us to control for unobserved heterogeneity that varies across countries. The fixed effects capture any time-invariant country-specific factors that may influence SDG performance, by including these fixed effects, we can obtain unbiased estimates of the effect of ODA net inflows on SDG performance. Specifically, we estimate the following SAC model:

2 $$\begin{aligned} \begin{aligned}&\textbf{SDG}_{i t}=\beta _1 ODA_{i t}+\rho \textbf{W} \textbf{SDG}_{t}+\textbf{X}_{i t} \boldsymbol{\beta }+\boldsymbol{\mu }_{i}+\boldsymbol{\mu }_{t}+\boldsymbol{\nu }_{i t} \\&\text {with}\quad \boldsymbol{\nu }_{i t}=\lambda \textbf{M} \boldsymbol{\nu }_{t}+\boldsymbol{\epsilon }_{i t} \end{aligned} \end{aligned}$$

where $\textbf{SDG}_{i t}$ is the SDG performance score for country *i* in year *t*, $ODA_{i t}$ is the ODA net inflows for country *i* in year *t*. The key parameters to be estimated are denoted by $\beta _1$, which indicate the impact of ODA on SDGs. The exogenous factors affecting the SDG performance are substituted by ${\boldsymbol{X}}_{i t}$. $\rho$ is the spatial autoregressive parameter, which measures the influence of the spatially lagged dependent variable on the current dependent variable. ${\boldsymbol{W}}\boldsymbol{SDG}_{t}$ is the spatially lagged SDG index accounting for various spatial dependencies with ${\boldsymbol{W}}$ defined as (n x n) spatial weight matrix. $\rho {\boldsymbol{W}}\boldsymbol{SDG}_{t}$ is the endogenous interaction effect. $\boldsymbol{\beta }$ is a vector of unknown parameters, which represent the coefficients of the covariates (independent variables).

We control for population because it captures structural factors relevant for SDG performance. Larger populations may provide greater human capital^52^, larger domestic markets, and economies of scale, but can also strain environmental resources^53^ and social services^54^. In addition, larger countries tend to receive higher absolute volumes of ODA^55^, which may further influence SDG outcomes. We also control for GDP per capita accounts for broad development capacity. Higher income levels are associated with stronger fiscal resources, improved infrastructure^56^, and greater access to health services^57^, all of which can facilitate SDG progress. Additionally, petroleum production captures natural resource endowments that may affect development trajectories. While resource revenues can support investment, resource dependence also carries risks such as institutional weakening, volatility, and environmental degradation^58^. This control accounts for these mixed theoretical mechanisms. UNGA voting alignment proxies for geopolitical ties and international political support. Greater alignment may be associated with higher development assistance^55,59^, e.g., or engagement in global governance, both of which may shape SDG-related capacities. However, alignment may also reflect geopolitical dynamics unrelated to development priorities. Lastly, we control for governance quality is essential for SDG achievement, as lower corruption is linked to more efficient government spending, better service provision, and stronger institutions^54,60^. External actors can also shape governance outcomes: Chinese aid projects have been shown to increase local corruption without improving short-term economic activity, while World Bank projects stimulate local economic activity without consistently increasing corruption^61^. Including corruption ensures that institutional quality is appropriately accounted for. The independent variables are further introduced in Section 3.

$\boldsymbol{\mu }_i$ is the fixed effect for country *i* and $\boldsymbol{\mu }_t$ is the fixed effect for the year *t*. $\boldsymbol{\nu }_{i t}$ is the spatially error term accounting for spatial correlation in the error term with ${\boldsymbol{M}}$ also defined as (n x n) spatial weight matrix. ${\boldsymbol{M}}$ is a matrix of spatial weights that equals to ${\boldsymbol{W}}$, which means that we consider the spatial autoregression of the error term to be consistent with that of the spatially lagged dependent variable. The influence of the spatial correlation in the error term is captured by $\lambda$. The standard assumptions, that $\epsilon _{i t} \sim N\left( 0, \sigma _\epsilon ^2\right)$ and $E\left( \epsilon _{i t} \epsilon _{j s}\right) =0 \text{ for } i \ne j \text{ or } t \ne s$, apply in this case.

To investigate heterogeneity in the ODA–SDG relationship, we incorporate a set of interaction terms capturing cultural, historical, and geographic dimensions that may condition the effectiveness of development assistance. First, we include an interaction between ODA and English-speaking countries to capture the role of language in facilitating cross-country engagement. A common language can reduce transaction costs in communication, contracting, and monitoring, thereby improving aid effectiveness. This builds on evidence showing that shared language statistically significantly increases international trade^62^ and that language barriers reduce bilateral exchange^63^. More broadly, cultural proximity, including shared language, has been shown to support greater economic interaction^64^ and trade intensity^65^. This interaction term therefore tests whether linguistic and cultural proximity enhance the development returns to ODA. Second, we examine whether religion shapes the relationship between ODA and SDG achievement. Religion has long been linked to economic and social development outcomes, although findings remain mixed. For example, one study^66^ shows that religion matters for explaining economic performance but does not find consistent effects for particular religions. Historical evidence from Prussia suggests that Protestant regions developed higher education levels than Catholic regions, which translated into long-term development gains^67^. By interacting ODA with indicators for the dominant religion (Christianity and Islam), we test whether cultural norms and value systems associated with major world religions influence how effectively external assistance translates into SDG progress. Third, we incorporate colonial history as an interaction factor, motivated by extensive research demonstrating that colonial legacies continue to shape contemporary economic, institutional, and political outcomes. For instance, one study documents the persistent negative effects of the slave trade on economic development^68^; another study shows that extractive colonial institutions have long-term impacts on economic performance^69^; and a further study demonstrates that colonial ties strongly influence ODA allocation^55^. Geography, often intertwined with colonial trajectories, likewise affects development indirectly through institutions and structural constraints^70^. Interacting ODA with colonial history allows us to capture whether historical political relationships condition the extent to which aid supports SDG advancement. Finally, we include interaction terms for structural geographic characteristics, such as whether a country is a Landlocked Developing Country (LLDC). LLDCs face substantially higher transport costs, reduced market access, and structural barriers to trade and economic diversification. These disadvantages may influence both the volume of ODA allocated and the effectiveness of aid in promoting sustainable development. By interacting ODA with an LLDC indicator, we test whether aid yields differential impacts in countries facing structural geographic constraints. Taken together, these interaction terms allow us to assess not only whether ODA contributes to SDG achievement on average, but also whether its impact varies systematically across cultural, historical, and geographic contexts.

In this paper, we focus on the fixed effect variant of the specification, which is a statistical technique used in panel data analysis. The reason for choosing this variant is that it incorporates the country and time fixed effect across the panel data, which is a crucial factor in controlling for the endogeneity due to the omitted variables. By including the country and time fixed effects, we can account for the unobserved heterogeneity that varies across countries but remains constant over time. This technique allows us to control for all time-invariant country-specific characteristics that may affect the outcome variable, such as cultural differences, historical events, institutional frameworks, and other unobserved factors. Thus, the fixed effect variant of the specification is an effective method to reduce bias and improve the accuracy of the estimation, and it is widely used in empirical research to address endogeneity issues in panel data analysis. Overall, by controlling for unobserved heterogeneity (country and time fixed effects) and potential spatial spillover effects (lagged spatial weight matrix), we can obtain more accurate estimates of the relationship between ODA and SDG performance, and provide valuable insights for policymakers aiming to promote sustainable development.

To estimate the model, we use a maximum likelihood approach, which allows us to obtain consistent estimates of the parameters, including the spatial autocorrelation parameter. We also conduct robustness checks to ensure the validity of our results (re-estimated the SAC model (equation (2)) with fewer control variables and for an extended group of countries). We rely on Stata to perform this analysis. Specifically, we utilize the xsmle package, which is a command for spatial analysis in Stata^71,72^. The xsmle package provides quasi-maximum likelihood estimation of a wide range of both fixed- and random-effects spatial models for balanced panel data. The xsmle package allows us to estimate models that account for both the spatial autocorrelation and heterogeneity that may be present in the data, and obtain accurate and reliable estimates of the parameters of interest^71^.

The SAC model is employed because it accommodates the two key sources of spatial dependence likely to arise in cross-country SDG performance. Specifically, the SAC specification captures endogenous spatial dependence through the spatially lagged dependent variable, as well as spatial correlation in the error term. This dual structure is particularly relevant in an international setting, where SDG outcomes may be shaped both by observable cross-border interactions and by unobserved regional shocks that exhibit spatial correlation^51^. By accounting for both channels, the SAC model offers a more comprehensive representation of the underlying spatial processes than single-equation alternatives.

We also examined alternative spatial specifications, including the Spatial Autoregressive (SAR) model^73^, the Spatial Error Model (SEM)^74^, and the Spatial Durbin Model (SDM)^75^. SAR and SEM capture only one dimension of spatial dependence, while SDM, although more flexible, may introduce unnecessary complexity and potential over-parameterization given our research focus^74^. We acknowledge that the SAC model is itself sensitive to the choice of spatial weight matrix and may not capture all forms of spatial heterogeneity. To address this concern, we evaluated several alternative spatial weight matrices and confirmed that the contiguity-based matrix used in the main analysis provides the best performance. In addition, we re-estimated the model using SAR, SEM, and SDM specifications as robustness checks.

### Reverse causality and the possible endogeneity

Endogeneity might pose a considerable concern, particularly in our context where reverse causality stands out as a potential source. Within this study, our primary focus revolves around the potential endogeneity associated with the ODA variable. There exists a likelihood of a two-way relationship between ODA and factors such as economic development (SDG 8). On one hand, donors might intentionally allocate more ODA to nations with higher per capita income, presuming these countries possess ’better’ economic policies and institutions. On the other hand, donors could also be inclined to provide greater ODA to poorer countries that struggle economically to progress without external assistance^34^.

The IV approach is widely used as a means to address endogeneity concerns, especially when associated with non-experimental data^45^. Through this, the aid variable can be purged from its correlation with the error term^34^. The interpretation of IVs estimates in terms of causality relies on the assumption that the only reason for any relationship between instruments and the dependent variable is the connection between instruments and the variable used the instrument for^76^. Hence, the IV method relies on the availability of a suitable instrument. We propose one instrument to examine the causal relationship between ODA on SDG performance: the lag of ODA received. The instrument must generate exogenous variation on ODA, which should otherwise be unrelated to SDG performance.

The lagged values of the identified potential endogenous variables are frequently utilized as instruments^34^. Employing the lag of ODA received as an IV assumes that past ODA receipts have an impact on current ODA allocation but do not directly affect SDG performance except through their influence on current ODA disbursement. Hence, the exclusion restriction for the suggested instruments might hold because the instrument should not have a direct impact on SDG performance. In summary, we use one instruments (lag of ODA received) to investigate the effects on SDG performance. Accordingly, in the first stage, we utilize the lagged ODA to estimate the current ODA, which is:

3 $$\begin{aligned} ODA_{it} = \alpha _1\, ODA_{it-1} + \textbf{X}_{it} + v_{it}, \end{aligned}$$

In the second stage, the predicted current ODA, denoted as ${\widehat{ODA}}_{it}$, is substituted into the equation (2) to address the endogeneity arising from the reciprocal causality between ODA allocation and SDG performance, which is:

4 $$\begin{aligned} \begin{aligned}&\textbf{SDG}_{i t} = \beta _1\, {\widehat{ODA}}_{it} + \rho \, \textbf{W}\, \textbf{SDG}_{t}+\textbf{X}_{i t} \boldsymbol{\beta }+\boldsymbol{\mu }_{i}+\boldsymbol{\mu }_{t}+\boldsymbol{\nu }_{i t}, \\&\text {with}\quad \boldsymbol{\nu }_{it} = \lambda \, \textbf{M}\, \boldsymbol{\nu }_t + \boldsymbol{\epsilon }_{it}, \end{aligned} \end{aligned}$$

## Data

We use the SDG indices developed in the SDR2022^7^, which provides a comprehensive assessment of country-level progress toward the 17 SDGs. The index includes 85 indicators covering 163 countries, and consists of one overall SDG index and 17 goal-specific indices^77^. The list of the countries is provided in Table A1 in Supplementary Material. Approximately two-thirds of the indicators come from official sources (e.g., UN agencies), and one-third from academic or non-governmental institutions. All indicators are normalized on a 0–100 scale using min-max scaling and aggregated with equal weights. External audits confirm the robustness and transparency of the index methodology^78^. To ensure cross-year comparability, the SDR2022 provides harmonized time series from 2000 to 2021 using consistent indicators and methods^7^. Table A2 summarizes the number of indicators included in each of the SDGs. The number of indicators varies considerably between goals, with SDG 2 (zero hunger) having the highest number of indicators (17) and SDG 10 (reduced inequalities) having only one. Given that ODA itself is one of several indicators of SDG 17, it is important to interpret the SDG 17 results with caution. However, the SDG 17 index is a highly multidimensional composite that extends far beyond ODA commitments. In addition to the ODA/GNI indicator (17.2.1), the goal includes indicators on domestic resource mobilization, remittances, debt sustainability, trade regimes, technology access, macroeconomic stability, policy coherence, use of country systems, multi-stakeholder partnerships, and statistical capacity, among others. As a result, the relationship between ODA and SDG 17 in our regressions should be interpreted with caution, as ODA represents only one element within SDG 17. Because SDG 17 includes many dimensions unrelated to ODA, our estimated coefficient may underestimate the true effect of ODA on the goal. In other words, this setup could lead to attenuation (downward) bias in the regression results.

The key control variables employed in the SAC model are the net ODA received (measured in constant 2020 US$) from 2000 to 2021 for each countries^79^. Furthermore, we have taken the potential impact of a country’s demographic factors and economic development on its ability to achieve the SDGs into consideration. Specifically, we have used the country’s total population, GDP per capita (measured in constant 2020 US$) obtained from World Development Indicators^80^, as well as petroleum production^81^ as control variables.

One study^82^ highlight﻿s that political institution matter when discussing the (economical) performance of a particular country. Hence, political preferences might explain the SDG performance. A common measure of the extent of country’s international political power is that other countries vote for it in the United Nations General Assembly (UNGA)^83^. For instance, one study^30^ highlight that similar UNGA voting pattern affect the allocation of US aid. Another study^84^ provides a detailed description of the methodology used to construct the UNGA vote Index.

We also include indicators that reflect the broad dimensions of governance, i.e., the control of corruption, as further control variables^85^. The control of corruption indicator was obtained from the Worldwide Governance Indicators (WGI)^80^. The WGI are based on over 30 underlying data sources reporting the perceptions of governance of a large number of survey respondents and expert assessments worldwide. Estimate of governance in standard normal units ranging from approximately -2.5 (weak) to 2.5 (strong) governance performance^86^.

To analyze the heterogeneous effect of ODA on SDG achievement, we incorporate several interaction terms with ODA. Specifically, we consider the cultural dimension and examine the interaction between ODA and the primary language spoken in a country, specifically English. Our objective is to investigate how the ease of communication between countries may lead to additional gains compared to those with language barriers. This idea is based on a meta-analysis on the relationship between language and international trade^62^. The authors highlight that a common language has a statistically significant increase on trade flows. Additionally, one study highlights that language barriers are statistically negatively associated with bilateral trade^63^. The list of countries and territories where English is a primary language are obtained from the Univesity of Groningen^87^. One study proves that cultural proximity positively affects trade volumes^64^. An additional study shows that countries with weaker ancestral relationships are less likely to trade with each other^65^. Second, we also investigate how a countries’ main religion, such as Christianity and Islam affect the ODA’s effect on the SDG performance. The religion data is obtained from^88^. One study investigates the relationship between religion and economic performance^66^ . The authors show that religion matters when explaining economic performance. However, the author cannot identify a robust pattern for particular religions. Another study highlights that the emergence of Protestantism in the late 19th century Prussia led to an increase in the education level in comparison to Catholic counties^67^. Therefore, language and religion are used as cultural proxies to investigate if the culture can explain SDG fulfillment as well as heterogeneous patterns.

We also consider the colonial history and geographic location as factors that may occur with the heterogeneous impact of our analysis. The colonial past impacts the current economic development of former colonies^68^. The author finds an inverse relationship between slave trade and economic development. One study shows that the colonial past are major determinants of ODA allocation^55^. Another study shows that the institutions created by the colonial ruler also affect the economic development of the former colony^69^. According to the authors, these institutions persist to the present. Hence, institutions created under colonial rule affect the income per capita of the former colony today^69^. Furthermore, one study shows that tropics, germs, and crops, as proxies for geography, affect economic development through institutions^70^. Hence, institutions, and not geography, that have been created over time (e.g., during the period of colonialism) might explain the patterns. Therefore, we use colonial history, measured as a dummy, times ODA as a interaction term to estimate the heterogeneous effect of political ties to explain SDG achievement. We also controlled for interaction terms between ODA and whether a country is a LLDC. We introduced a dummy variable with a value of 1 indicating that a country is a LLDC, and a value of 0 indicating that a country is not a LLDC. The list of LLDC are obtained from UN, Office of the High Representative for the Least Developed Countries, Landlocked Developing Countries and Small Island Developing States^89^.

The key variables for this research are SDG Indices and ODA received. Table 1 shows the summary statistical results for control variables used in the SAC model from year 2000 to 2021. The table is divided this study. Due to the data avaliablity, Columns (1)-(5) summary 158 countries with SDG scores included in SDR2022 and the other for countries that have received ODA. There are some counties that have several missing data problem, such as Venezuela, Somalia, South Sudan, Djibouti, and Serbia, we dropped these countries from our sample. Some countries, such as Afghanistan (only with missing values in year 2000 and year 2001) and Syria (only with missing values in year 2021) have slight missing data problem, we use the interpolation method to complete missing year data^90^. In addition, there are 23 developed countries of the Global North in the sample that do not receive ODA between 2000 and 2021 and therefore we summary the characteristics without those developed countries in Columns (6)-(10), see Table A3 in Supplementary Material for 23 developed countries. As a result, a balanced panel data set comprising of 135 countries from the year 2000 to 2021 are utilized for the analysis in Section 4.3.

The first set of variables are ODA received (in million US$), population (in million), GDP per capita (in thousand US$), petroleum production (in million tons), UNGA vote Index, and corruption Index. Comparing the means between all countries and countries that have received ODA, we can see that the mean value for ODA received is higher for the latter group, indicating that ODA is targeted towards countries that need it the most. Population and petroleum production have similar means for both groups, while the mean GDP per capita is higher for all countries than for countries that have received ODA, which suggests that the ODA is focused on poorer countries. The UNGA vote Index and corruption Index have negative means for both groups, which implies that countries tend to vote against each other and corruption is a common problem not specifically in the Global South.

The second set of variables are used in interaction terms, including low income countries, SSA, LLDC, English, Christian, Islam, and colonial history. The mean values for these variables between all countries and countries that have received ODA are generally similar, with some small variations. For example, the mean values of low income country and SSA are slightly higher for countries that have received ODA, while the mean values of LLDC and colonial history are slightly higher for all countries. These variables are used in interaction terms to investigate the effectiveness of ODA in different country groups.

**Table 1:** Summary statistic

	Entire sample of 158 countries					ODA recipient countries				
	N	Mean	SD	Min.	Max.	N	Mean	SD	Min.	Max.
	(1)	(2)	(3)	(4)	(5)	(5)	(7)	(8)	(9)	(10)
*Control variables*										
ODA received (million USD)	3476	693.48	1236.26	0.00	26340.29	2970	811.62	1301.11	0.00	26340.29
Population (million)	3476	43.53	149.30	0.14	1412.36	2970	44.23	159.22	0.14	1412.36
GDP per capita (thousand USD)	3466	12.80	18.22	0.26	112.42	2960	7.02	9.89	0.26	73.49
Petrolium production (million tons)	3476	24.15	76.56	0.00	780.61	2970	21.89	70.20	0.00	586.60
UNGA vote Index	3450	-0.11	0.86	-2.11	2.94	2946	-0.32	0.73	-2.11	2.60
Corruption Index	3318	-0.07	1.00	-1.78	2.46	2835	-0.36	0.73	-1.78	2.30
*Variables used in interaction terms*										
Low income country (1=yes)	3476	0.42	-	0.00	1.00	2970	0.50	-	0.00	1.00
Sub-Saharan Africa (SSA) (1=yes)	3476	0.25	-	0.00	1.00	2970	0.30	-	0.00	1.00
Landlocked Developed Countries (LLDC) (1=yes)	3476	0.20	-	0.00	1.00	2970	0.23	-	0.00	1.00
English (1=yes)	3476	0.16	-	0.00	1.00	2970	0.14	-	0.00	1.00
Christian (1=yes)	3476	0.63	-	0.00	1.00	2970	0.58	-	0.00	1.00
Islam (1=yes)	3476	0.27	-	0.00	1.00	2970	0.32	-	0.00	1.00
Colonial history (1=yes)	3476	0.56	-	0.00	1.00	2970	0.65	-	0.00	1.00

Notes: To increase the transparency, the summary statistics, display the continuous values. The continuous variables ODA received, population, GDP per capita and petroleum production are used in the logarithmic form in the SAC model. Columns (1)–(5) include data for all countries featured in the Sustainable Development Report with no missing values^7^, while Columns (6)–(10) exclude 23 developed countries that did not receive any ODA between 2000 and 2021

## Results

### Changes in SDG indices and ODA

**Fig. 2:**
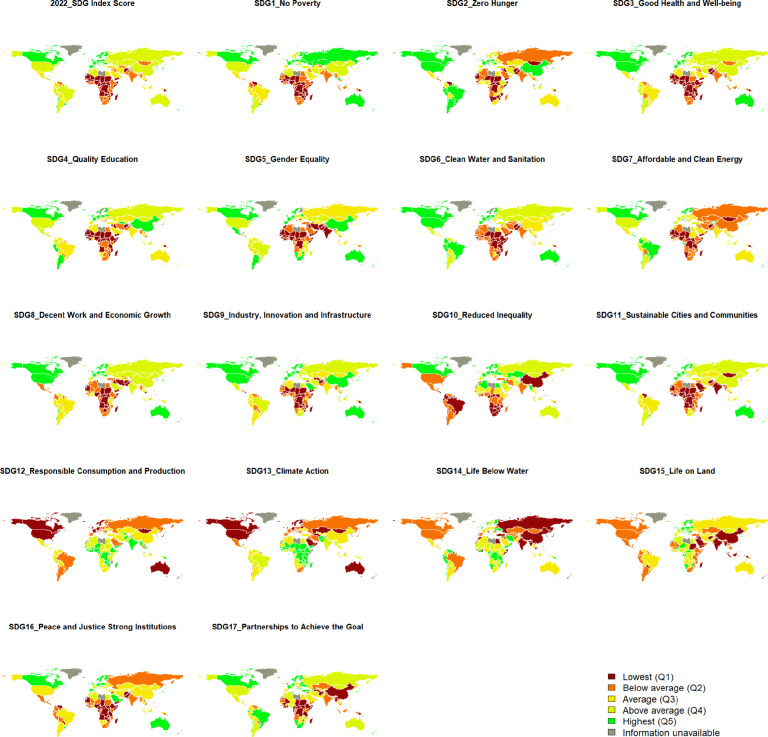
Traffic light for World SDGs Index with 5 quantiles. Notes: Countries or regions marked in red means those countries are located in the lowest 20 percent compared to other countries or regions, while green means those counties or regions have good performances in SDGs Index. N=163. Source: the SGDs Index comes from the 2022 SDG report issued by EU and the world shape file comes from^91^. Source: Own visualization based on the software R, Version: 2022.02.0

The global SDGs performance Index in the latest period, which is 2022, are illustrated in Figures 2. These Figures in 2 suggest that there are clear regional clusters in the distribution of scores. Specifically, the countries with higher overall SDG Index scores are predominantly located in the continents of North and South America, as well as Europe. Conversely, countries with lower overall scores are concentrated in the African continent.

However, when we examine the performance of individual countries in relation to specific SDGs, we observe a nuanced picture. On the one hand, with regard to SDG 1 (no poverty), which focuses on eradicating poverty, the majority of countries with lower SDG 1 (no poverty) Index Scores are located in SSA. On the other hand, when we turn our attention to SDG 12 (responsible consumption), SDG 13 (climate action), SDG 14 (life below water), and SDG 15 (life on land), which are related to the environmental goals, the performance of the African countries is relatively better in comparison to other world regions. For example, countries in North and South America face relatively more considerable challenges in these areas. The clustering effect of the SDG index across different regions may indicate the influence of regional factors that impact the performance of the SDGs. These could include factors such as economic development, access to resources, and regional policies.

**Fig. 3:**
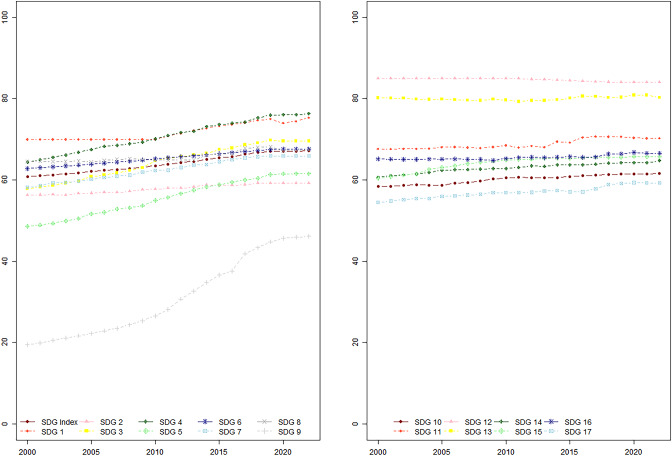
SDG index scores changing trend averaged over all 163 countries 2000 to 2022). Source: Own visualization based on the software R, Version: 2022.02.0

Figure 3 shows the annual changing trend for overall SDG index and 17 separate goals from the years 2000 to 2022. Due to data availability, we include all available data up to the year 2022, although we only cover the period from 2000 to 2021 in our SAC model. Table A4 in Supplementary Material provides a descriptive statistical table of the annual changing trend of the SDGs as a supplement to this figure. The overall SDG index score measures the progress of countries towards achieving the SDGs, while the individual SDG scores indicate the performance of countries in specific areas. From Figure 3, it becomes clear that the degree to which each SDG target has been achieved varies considerably. Overall, the SDG index scores improved from 60.84 in 2000 to 67.17 in 2022, indicating that there is still substantial progress to be made in meeting the SDGs. When we examine the performance of individual SDG targets, we observe that SDG 12 (responsible consumption and production), SDG 13 (climate action), SDG 4 (quality education), and SDG 1 (no poverty) have achieved relatively better results compared to other SDGs. However, the scores of other SDGs are relatively lower, indicating the need for increased attention and focus in these areas, for example, the SDG 9 (industry, innovation, and infrastructure) had the lowest score. This suggests that certain SDGs may require more attention and resources to be achieved as planned by 2030 compared to others. Despite the UN’s recognition that all 17 SDG targets should be equally prioritized, the varying degrees of progress in achieving these targets highlight the considerable differences in the implementation of different goals. Overall, there were improvements in most of the SDGs over the years.

### Spatial correlations of SDGs: Moran’s I

Table 2 shows the results for Moran’s I test for the overall SDG index and 17 separate goals’ Index based on the geographical weighting matrix. As noted earlier, the values of global Moran’s I range from ”-1” to ”1”. If Moran’s I is above 0, there is positive spatial correlation between SDGs score. The values of global Moran’s I range from 0.159 (SDG 14 - life below water) to 0.794 (SDG 1 - no poverty) indicating a positive spatial correlation for all 17 SDGs as well as the SDG index score.

Table of critical values for Moran’s I at various levels of statistically significance and sample sizes are available in statistical textbooks^92,93^. The critical values of Moran’s I for a 95% statistically significance level at sample size 163 is 0.154. In the context of SDGs, if the Moran’s I is found to be greater than 0.154, it can be concluded that there is a statistically significant level of spatial autocorrelation present in the SDG index score. The Moran’s I is greater than 0.154 for all SDGs as well as for the 2022 SDG index score. Hence, the results from Table 2 suggest that the values of the SDGs Index are not randomly distributed across space, but are instead influenced by the situation of neighboring locations.

The value of the 2022 SDG index score is 0.733, which is relatively close to 1. As larger value highlight a stronger correlation, this indicates a strong positive spatial correlation for the SDG index score. Focusing on separate SDGs, we observe a heterogeneity in the magnitude of the positive spatial correlation across different SDGs. The strongest positive spatial correlation exists for SDG 1 (no poverty), SDG 3 (good health and well-being), SDG 6 (clean water and sanitation), and SDG 10 (reduced inequalities) with values larger than 0.651, while the relatively weakest positive spatial correlation exist for SDG 2 (zero hunger), SDG 13 (climate action), SDG 14 (life below water), SDG 15 (life on land), and SDG 17 (partnership for the goals) with positive values ranging from 0.159 to 0.356.

Our analysis reveals spatial autocorrelation in the SDG index, indicating that countries’ performances in achieving the SDGs are geographically clustered rather than randomly distributed. Both the traffic-light visualization and Moran’s I results identify clear country clusters. These spatial clusters suggest shared challenges, strengths, and priorities among neighboring countries, highlighting potential benefits from coordinated actions and regional collaborations to enhance progress toward sustainable development.

**Table 2:** Moran I test for SDGs Index in 2022

	(1)	(2)
	Moran I statistic	*p*-value
SDG index score	0.733	0.000
SDG 1 Score	0.794	0.000
SDG 2 Score	0.329	0.000
SDG 3 Score	0.688	0.000
SDG 4 Score	0.651	0.000
SDG 5 Score	0.548	0.000
SDG 6 Score	0.708	0.000
SDG 7 Score	0.419	0.000
SDG 8 Score	0.480	0.000
SDG 9 Score	0.529	0.000
SDG 10 Score	0.671	0.000
SDG 11 Score	0.573	0.000
SDG 12 Score	0.543	0.000
SDG 13 Score	0.356	0.000
SDG 14 Score	0.159	0.002
SDG 15 Score	0.350	0.000
SDG 16 Score	0.482	0.000
SDG 17 Score	0.316	0.000
Observations	163	

The Moran’s I is calculated based on basic binary geographical weighting matrix. The SDG index score utilized in the table represents the most recent data available, specifically from the year 2022.

### Heterogenous effects of ODA on SDG goals

This section presents the results of the SAC models, incorporating county and year fixed effects to assess the impact of ODA received on SDG performance. As previously noted, endogeneity may arise due to reverse causality. To address this, lagged ODA received are used as an IV in the first stage. The second-stage SAC model then replaces observed ODA received with predicted values to mitigate endogeneity. Table A6 in Supplementary Material indicates that lagged ODA received are statistically significantly correlated with observed ODA received, supporting their validity as an IV. Accordingly, the following analysis employs predicted ODA received to ensure unbiased estimation.

Table 4 shows the results for the impact of net ODA received on ovearall SDG index as well as 17 separate SDGs indices. ODA received have a statistically significant negative impact on the overall SDG index. We only find statistically significant positive effects of ODA on limited SDGs, i.e., SDG 1 (no poverty), SDG 2 (zero hunger), SDG 6 (clean water and sanitation). However, statistically significant negative effects are observed on overall SDG index, SDG 8 (decent work and economic growth), SDG 9 (industry, innovation, and infrastructure), SDG 15 (life on land), and SDG 16 (peace, justice and strong institutions). This result highlights the heterogeneity of ODA’s impact on different SDGs. It suggests that ODA, as a common and effective means of economic aid to developing countries, may have limited assistance for certain SDGs. For instance, aid projects may focus more on reducing hunger and poverty, rather than gender equality and other SDGs. This finding is consistent with previous literature^42^, which also found that donor countries prioritize the basic needs of developing countries, such as poverty and hunger, when providing aid.

The positive impacts on SDG 1 (no poverty), SDG 2 (zero hunger), and SDG 6 (clean water and sanitation) are intuitive given that ODA is often directed toward basic human needs. Donor countries have historically prioritized funding for poverty alleviation, food security, and essential services in developing nations^94^. This aligns with a previous finding^42^, who observed that donors tend to target aid in line with basic MDGs such as reducing extreme poverty and hunger. Similarly, a study^94^ documented that a large share of development finance is allocated to health (related to SDG 3) and hunger (SDG 2), reflecting donors’ emphasis on fundamental well-being. The positive coefficient for SDG 6 (clean water and sanitation) suggests that aid has also been effective in expanding access to clean water and sanitation infrastructure, likely through funding of wells, water systems, and sanitation projects in underserved regions. In sum, the significant gains in these specific goals indicate that ODA can be effective in improving basic living conditions – a result consistent with micro-level evidence of successful aid-funded interventions in health, nutrition, and infrastructure. These targeted improvements, however, do not automatically translate into broad sustainable development progress, as evidenced by the neutral or negative effects on other SDGs.

Additionally, Figure A1 in Supplementary Material shows the allocation of the ODA. Specifically, SDG 3 (good health and well-being) is the most targeted SDG, followed by SDG 2 (zero hunger), and SDG 16 (peace, justice and strong institutions). This is not surprising, as health is a fundamental component of human well-being, and improving access to healthcare is crucial for achieving sustainable development. Similarly, addressing hunger and promoting peace and justice are essential for creating a stable and equitable society, which in turn is essential for achieving sustainable development. However, it is important to note that higher ODA allocations, as shown also in Figure A1, do not necessarily translate into uniformly positive or statistically significant coefficient estimates across SDGs. Descriptive prioritization alone cannot predict econometric outcomes because SDGs differ substantially in their scope, complexity, financing needs, and the measurability of short- and medium-term progress. These structural differences may imply that even SDGs receiving above-average ODA may exhibit weaker, insignificant, or even negative associations in the regression results.

Our results show that ODA is associated with poorer performance on SDG 8 (decent work and economic growth) and SDG 9 (industry, innovation, and infrastructure). A key explanation is the macroeconomic distortions linked to large aid inflows. Extensive evidence documents a “Dutch disease” effect, whereby aid-induced real exchange rate appreciation reduces export competitiveness and slows industrial development^35^. One study similarly find that aid-dependent countries experience systematically lower growth in manufacturing sectors, attributable to currency appreciation and weakened incentives for private investment^36^. These dynamics help explain why higher ODA in our analysis correlates with slower progress in employment and industrial capacity.

We also find negative effects of ODA on SDG 15 (life on land) and SDG 16 (peace, justice and strong institutions). Prior studies offer a rationale for these results: environmental objectives have often been secondary to short-term development priorities, and aid-funded projects in agriculture or infrastructure can inadvertently contribute to deforestation or habitat loss when safeguards are weak^37^. At the same time, the “aid–institutions paradox” suggests that large aid inflows may undermine governance by reducing accountability, encouraging rent-seeking, and weakening bureaucratic capacity; empirical analyses^38,39^ document declines in governance and democratic quality in highly aid-dependent countries. These mechanisms help explain why higher ODA is associated with poorer outcomes in environmental sustainability and institutional performance in our results.

Overall, these patterns underscore that ODA’s contributions to sustainable development are uneven: ODA appears to bolster progress on poverty, hunger, and water access, but may hamper or have no effect on several other goals, including economic growth, industrial development, environmental conservation, and institutional quality. This pattern echoes a literature emphasizing trade-offs among the SDGs. For example, one study finds that global gains in poverty reduction often occur at the expense of other critical SDGs, especially those associated with the environment^3^. Likewise, one study analyses the interlinkages for different sets of SDGs and argues that the SDG 1 (no poverty) and 2 (zero hunger) are consistency far downstream of the other goals, meaning that progress on the SDG 1 (no poverty) and 2 (zero hunger) often reaps the rewards and benefits of progress in other areas^23^. However, SDGs 12 (sustainable consumption and production) and 17 (partnerships) consistently appear far upstream, meaning that these are goals that enable progress. Meanwhile, studies highlight that trade-offs and complementarities among the different SDGs exist^3,24^. These findings caution against assuming that progress in one SDG will automatically generate positive spillovers to others, and instead underscore the importance of accounting for goal conflicts when assessing ODA’s role in sustainable development.

Additionally, while we observe statistically significant positive associations between ODA and SDG 1 (no poverty), SDG 2 (zero hunger), and SDG 6 (clean water and sanitation), these effects should not be interpreted as a mechanical consequence of ODA prioritization alone. Although SDG 3 (good health and well-being) and SDG 16 (peace, justice and strong institutions) also receive substantial ODA volumes, progress in these areas is typically more complex, governance-intensive, and slower to materialize. In contrast, interventions linked to SDG 1 (no poverty), SDG 2 (zero hunger), and SDG 6 (clean water and sanitation), such as agricultural productivity support, social protection programmes, and water and sanitation infrastructure, tend to involve more clearly defined, actionable, and measurable project types with shorter causal chains. This difference in scope and implementation complexity helps explain why statistically significant associations emerge for these goals but not for SDG 3 or SDG 16. This interpretation is consistent with recent analyses by the International Financial Corporation^95^ and UN^96^ that highlight persistent financing gaps and structural constraints in health and governance sectors, which limit the short- and medium-term impact of ODA despite substantial commitments. Our findings therefore align with the broader literature showing considerable variation in ODA effectiveness across sectors, reinforcing the need to interpret SDG-specific coefficients in light of sectoral characteristics and implementation realities.

As for the control variables, the population has a statistically significant positive relationship with more than half of the SDGs, as well as GDP per capita, while petroleum production, UNGA vote Index, and corruption Index do not show statistically significant effect on most of the SDGs. Overall, the results suggest that both population and GDP per capita play a role in achieving the SDGs. Countries with larger populations and higher GDP per capita are generally more likely to achieve the SDGs. As for the spatial parameters, Rho measures the influence of the spatially lagged dependent variable on the current dependent variable, while Lambda measures the influence of the spatial correlation in the error term. Our findings indicate that both Rho and Lambda have statistically significant effects on eleven SDGs and seven SDGs respectively. This result indicates the presence of spatial autocorrelation and validates the use of spatial autocorrelation models.

Several robustness checks were conducted. First, we performed a diagnostic assessment by applying Moran’s I tests to the residuals of all SDG equations to determine whether any spatial autocorrelation remained after model estimation. For all 18 SDG indicators, the Moran’s I statistics are small and statistically insignificant, indicating that the SAC specification successfully captures spatial dependence. This confirms that the residuals behave as expected under correct model specification and supports the robustness of our empirical findings (see Table A7 in the Supplementary Material). Second, we assessed methodological robustness by estimating three alternative spatial models, SAR, SEM, and SDM. These models isolate different dimensions of spatial dependence and thus serve as informative complements to the SAC specification. As shown in Tables A8–A10, the core results remain qualitatively and quantitatively consistent across all three models. Third, we re-estimated the SAC model with fewer control variables (see Table A11 in Supplementary Material) and further excluded GDP per capita variables (see Table A12 in Supplementary Material). The results remain robust to our preferred and comprehensive model in Table 4. Finally, Table A13 in Supplementary Material shows the results for an extended group of countries. In this model, we also include countries in the estimation that did not receive ODA and re-estimate the equation (3). Based on this broad and diverse sample, the positive and statistically significant effect observed in the previous models for SDG 1 (no poverty) and SDG 2 (zero hunger) remains robust. However, we would like to emphasize that the estimates that include only countries that receive ODA are preferable, as countries that do not receive ODA have a net ODA value of 0, which biases the result downward.

### Heterogeneous effects of ODA by different groups of countries

Table 5 presents the heterogeneous effects of ODA on SDG performance of different groups of countries. The first set of results considers Low-Income Countries (LICs) and finds that the interaction effect between ODA and low-income country is positive and statistically significant for SDG 1 (no poverty), indicating that the positive effect of ODA on progress towards achieving this goal is greater for low-income countries compared to middle-income and high-income countries. On the other hand, the interaction effect is negative and statistically significant for SDG 9 (industry, innovation, and infrastructure) and SDG 16 (peace, justice and strong institutions), suggesting that the negative effect of ODA on progress towards achieving this goal is greater for low-income countries. Overall, the results suggest that the effect of ODA on progress towards achieving SDGs is heterogeneous across countries and goals, and low-income countries may benefit more from receiving ODA for some goals than other countries. Additionally, the second set of results examines the interaction effect of ODA and SSA, the third set of the results examines the interaction effect of ODA and LLDC. The results indicate that the no greater positive effects for SSA countries compared to other countries. While the positive effect of ODA on progress towards achieving SDG 2 (zero hunger) is greater for LLDC countries compared to other countries.

As for the cultural aspect, the fourth set of results examines the interaction effect of ODA and English-speaking countries, the fifth set of the results examines the interaction effect of ODA and Christian Countries, and the sixth set of the results examines the interaction effect of ODA and Islam Countries. The results show that the interaction between ODA and English-speaking has a positive and statistically significant effect on over all SDG index score, SDG 12 (responsible consumption and production), SDG 14 (life below water) and SDG 15 (life on land). However, the interaction effect is statistically significant different for predominantly Christian countries, where the interaction term between ODA and Christian is only positive and statistically significant in SDG 5 (gender equality), but negative and statistically significant in overall SDG index, SDG 6 (clean water and sanitation), SDG 11 (sustainable cities and communities), SDG 15 (life on land). This suggests that predominantly English-speaking countries benefit more from receiving ODA than non-English-speaking countries, whereas predominantly Christian countries do not display comparable advantages. The interaction term between ODA and Islam also has a statistically significant effect on several SDGs, indicating the importance of cultural differences. The effect of the interaction term is statistically significantly negative for SDG 5 (gender equality), SDG 9 (industry, innovation, and infrastructure), and SDG 17 (partnerships for the goals), but positive for SDG 13 (climate action), and SDG 15 (life on land).

The last set of results examines the interaction effect of ODA and countries’ colonial history. The interaction term between ODA and colonial history is statistically significantly negative for SDG 9 (industry, innovation, and infrastructure), but positive for SDG 2 (zero hunger), SDG 3 (good health and well-being), SDG 6 (clean water and sanitation), and SDG 7 (affordable and clean energy). These findings suggest that the effectiveness of ODA in promoting specific SDGs varies depending on whether a country is a former colony. For the remaining SDGs, however, the interaction effect between ODA and colonial history is not statistically significant, indicating no substantial difference in the impact of ODA between former colonies and non-colonies.

Overall, through the interaction term between ODA and each country group, Table 5 indicates the heterogeneous effect of ODA on the SDGs across different groups of countries. The results suggest that the effect of ODA on the SDGs varies statistically significant across country groups, especially for SDG 1 (no poverty) and SDG 2 (zero hunger). The heterogeneity of ODA’s impact on SDGs also reflects that the implementation of SDGs may have a prioritization order, despite the UN’ repeated declaration that the 17 SDGs should be treated equally, and all countries should promote them in a balanced manner. However, in reality, the implementation of SDGs may not occur simultaneously, and some goals may be achieved before others. Therefore, ODA’s allocation may also follow the order of SDG implementation, with more funds being distributed to SDG 1 (no poverty) and SDG 2 (zero hunger).

Furthermore, the heterogeneity of ODA’s impact on SDGs reflects the limited scope of aid provided in the form of ODA. Apart from specific SDGs, ODA has not demonstrated a statistically significant role in promoting the implementation of other SDGs, such as gender equality and environmental protection. Therefore, the scope and form of ODA may need further improvement to better align with the broader range of SDGs (Table 3).

**Table 3:** Impact of ODA on SDG index scores with SAC model

	(1)	(2)	(3)	(4)	(5)	(6)	(7)	(8)	(9)	(10)
	SDG index score	SDG1	SDG2	SDG3	SDG4	SDG5	SDG6	SDG7	SDG8	SDG9
ODA received (log)	-0.162**	0.525***	0.248**	-0.031	0.195	0.054	0.308***	0.292	-0.314***	-2.094***
	(0.075)	(0.124)	(0.097)	(0.097)	(0.247)	(0.197)	(0.078)	(0.177)	(0.106)	(0.457)
Population (log)	2.329**	4.771**	4.499**	7.390***	13.799***	2.700	2.658**	5.521**	2.436**	-5.551
	(0.947)	(2.081)	(1.937)	(1.670)	(5.011)	(3.380)	(1.148)	(2.550)	(1.203)	(7.385)
GDP per capita (log)	2.549***	9.365***	5.879***	4.383***	7.465***	1.170	3.524***	1.889	2.072***	-0.510
	(0.920)	(1.689)	(1.458)	(1.397)	(2.787)	(1.800)	(0.939)	(1.862)	(0.717)	(2.815)
Petrolium (log)	0.001	-0.314	-0.022	-0.109	0.659	-0.025	-0.161	0.019	0.131	0.730*
	(0.087)	(0.403)	(0.234)	(0.146)	(0.477)	(0.311)	(0.142)	(0.282)	(0.119)	(0.400)
UNGA vote index	-0.233	0.833	0.170	-0.572	0.652	-1.437	0.501	0.390	-0.478	0.891
	(0.217)	(0.699)	(0.418)	(0.495)	(1.023)	(0.890)	(0.358)	(0.630)	(0.302)	(0.925)
Corruption index	0.656**	0.798	-0.070	1.647***	0.618	1.357	-0.716*	0.728	0.466	2.205
	(0.299)	(0.871)	(0.668)	(0.621)	(1.291)	(0.939)	(0.402)	(1.219)	(0.460)	(1.473)
Spatial										
$\rho$	0.057	0.129***	0.088**	0.142***	0.111***	-0.099	0.131***	-0.021	0.085**	0.091
	(0.083)	(0.020)	(0.039)	(0.020)	(0.032)	(0.064)	(0.022)	(0.108)	(0.040)	(0.066)
$\lambda$	0.007	-0.148***	-0.059	-0.101***	-0.117***	0.111***	-0.172***	0.037	-0.076	0.000
	(0.087)	(0.038)	(0.050)	(0.038)	(0.045)	(0.030)	(0.038)	(0.091)	(0.049)	(0.080)
$R^2$	0.225	0.147	0.112	0.000	0.005	0.007	0.052	0.004	0.046	0.045
Country FE	Yes	Yes	Yes	Yes	Yes	Yes	Yes	Yes	Yes	Yes
Year FE	Yes	Yes	Yes	Yes	Yes	Yes	Yes	Yes	Yes	Yes
Observations	2,835	2,835	2,835	2,835	2,835	2,835	2,835	2,835	2,835	2,835

Standard errors in parentheses. * $p<0.10$, ** $p<0.05$, *** $p<0.01$. We excluded 23 countries that do not receive ODA from this sample and five countries due to data availability (Venezuela, Somalia, South Sudan, Djibouti, and Serbia). This also explains the different observations compared to the summary statistic

**Table 4:** Impact of ODA on SDG index scores with SAC model

	(11)	(12)	(13)	(14)	(15)	(16)	(17)	(18)
	SDG10	SDG11	SDG12	SDG13	SDG14	SDG15	SDG16	SDG17
ODA received (log)	-0.054	-0.237	0.035	-0.130	-0.255	-1.239***	-0.301*	0.030
	(0.158)	(0.229)	(0.026)	(0.119)	(0.211)	(0.334)	(0.166)	(0.144)
Population (log)	-2.837	8.499**	1.305***	-0.638	-3.279	-5.457	-1.567	-0.410
	(2.103)	(3.940)	(0.390)	(1.254)	(2.583)	(4.137)	(1.692)	(1.829)
GDP per capita (log)	-0.907	8.692***	-0.200	-3.830***	-4.234***	-0.295	2.080	0.356
	(1.065)	(2.108)	(0.197)	(1.144)	(1.246)	(1.421)	(1.404)	(1.583)
Petrolium (log)	0.457***	-0.124	-0.093	-0.409	-0.253	-0.450***	0.189	-0.006
	(0.172)	(0.314)	(0.073)	(0.255)	(0.163)	(0.174)	(0.245)	(0.198)
UNGA vote index	-1.281*	-1.299	0.003	0.243	-0.006	0.129	-0.004	-0.519
	(0.657)	(0.838)	(0.052)	(0.254)	(0.411)	(0.465)	(0.475)	(0.623)
Corruption index	0.601	0.765	-0.116	-0.315	-0.561	1.168*	1.604***	0.795
	(0.879)	(0.935)	(0.085)	(0.569)	(0.792)	(0.684)	(0.579)	(0.780)
Spatial								
$\rho$	0.119***	0.020	0.004	0.056***	0.029	-0.018	0.089***	0.059
	(0.026)	(0.181)	(0.017)	(0.021)	(0.077)	(0.043)	(0.024)	(0.058)
$\lambda$	-0.126***	0.044	0.032	-0.016	-0.059	0.039	-0.049	0.008
	(0.046)	(0.182)	(.)	(0.028)	(0.087)	(0.034)	(0.030)	(0.066)
$R^2$	0.017	0.057	0.294	0.452	0.009	0.055	0.443	0.040
Country FE	Yes	Yes	Yes	Yes	Yes	Yes	Yes	Yes
Year FE	Yes	Yes	Yes	Yes	Yes	Yes	Yes	Yes
Observations	2835	2835	2835	2835	2835	2835	2835	2835

Standard errors in parentheses. * $p<0.10$, ** $p<0.05$, *** $p<0.01$. We excluded 23 countries that do not receive ODA from this sample and five countries due to data availability (Venezuela, Somalia, South Sudan, Djibouti, and Serbia). This also explains the different observations compared to the summary statistic

**Table 5:** Heterogeneous impact of ODA on SDG index scores with SAC model

	(1)	(2)	(3)	(4)	(5)	(6)	(7)	(8)	(9)	(10)
	SDG index score	SDG1	SDG2	SDG3	SDG4	SDG5	SDG6	SDG7	SDG8	SDG9
Low Income Countries										
ODA received (log)	-0.136*	0.407***	0.237**	-0.013	0.263	0.131	0.272***	0.327*	-0.286**	-1.786***
	(0.071)	(0.120)	(0.097)	(0.087)	(0.209)	(0.190)	(0.070)	(0.168)	(0.112)	(0.438)
ODA received (log) * Low Income Country	-0.181	1.535**	0.100	-0.138	-0.771	-0.538	-0.090	-0.219	-0.224	-2.066***
(Std. Err.)	(0.223)	(0.619)	(0.291)	(0.349)	(0.920)	(0.505)	(0.214)	(0.594)	(0.222)	(0.637)
Sub-Saharan Africa (SSA)										
ODA received (log)	-0.158**	0.528***	0.248**	-0.056	0.170	0.036	0.341***	0.311*	-0.330***	-1.978***
	(0.076)	(0.125)	(0.097)	(0.091)	(0.249)	(0.196)	(0.081)	(0.175)	(0.106)	(0.458)
ODA received (log) * SSA	-0.071	-0.047	-0.001	0.500	0.030	0.462	-0.538	-0.626	0.280	-1.495**
	(0.152)	(0.489)	(0.331)	(0.386)	(0.727)	(0.528)	(0.332)	(0.714)	(0.247)	(0.715)
Landlocked Developed Countries (LLDC)										
ODA received (log)	-0.169**	0.465***	0.196**	-0.051	0.178	-0.001	0.293***	0.286*	-0.306***	-1.975***
	(0.074)	(0.129)	(0.095)	(0.096)	(0.247)	(0.191)	(0.072)	(0.168)	(0.107)	(0.453)
ODA received (log) * LLDCs	0.165	1.716	0.952*	0.467	0.110	0.840	-0.212	0.496	-0.181	-2.377**
	(0.372)	(1.364)	(0.567)	(0.714)	(1.412)	(0.993)	(0.564)	(1.180)	(0.404)	(1.092)
English										
ODA received (log)	-0.181**	0.501***	0.245**	-0.029	0.157	0.036	0.288***	0.290	-0.309***	-2.124***
	(0.078)	(0.120)	(0.095)	(0.096)	(0.244)	(0.198)	(0.074)	(0.180)	(0.107)	(0.466)
ODA received (log) * English	0.315*	0.475	0.048	-0.038	0.852	0.304	0.065	-0.036	-0.115	0.772
	(0.177)	(0.499)	(0.349)	(0.411)	(0.710)	(0.529)	(0.330)	(0.507)	(0.280)	(0.806)
Chrstian										
ODA received (log)	-0.073	0.555***	0.322***	0.056	0.293	-0.312	0.401***	0.386*	-0.353***	-1.890***
	(0.087)	(0.184)	(0.124)	(0.134)	(0.361)	(0.237)	(0.090)	(0.201)	(0.117)	(0.422)
ODA received (log) * Christian	-0.136*	-0.031	-0.105	-0.120	-0.161	0.548***	-0.118*	-0.152	0.058	-0.337
	(0.079)	(0.147)	(0.106)	(0.105)	(0.258)	(0.204)	(0.071)	(0.161)	(0.098)	(0.586)
Islam										
ODA received (log)	-0.156*	0.456***	0.220**	-0.060	0.319	0.251	0.287***	0.269	-0.269**	-1.658***
	(0.083)	(0.136)	(0.099)	(0.091)	(0.210)	(0.179)	(0.081)	(0.185)	(0.115)	(0.554)
ODA received (log) * Islam	-0.029	0.481	0.143	0.158	-0.754	-0.944**	0.116	0.066	-0.217	-1.740**
	(0.192)	(0.418)	(0.257)	(0.269)	(0.721)	(0.371)	(0.168)	(0.316)	(0.203)	(0.723)
Colonial history										
ODA received (log)	-0.174**	0.396***	0.141*	0.029	0.136	0.145	0.204***	0.084	-0.340***	-1.691***
	(0.084)	(0.106)	(0.079)	(0.085)	(0.221)	(0.219)	(0.065)	(0.161)	(0.117)	(0.480)
ODA received (log) * Colony	0.024	0.399**	0.236**	-0.146	0.148	-0.239	0.188*	0.463*	0.061	-0.828**
	(0.100)	(0.201)	(0.120)	(0.131)	(0.338)	(0.234)	(0.099)	(0.264)	(0.126)	(0.408)
Country FE	Yes	Yes	Yes	Yes	Yes	Yes	Yes	Yes	Yes	Yes
Year FE	Yes	Yes	Yes	Yes	Yes	Yes	Yes	Yes	Yes	Yes
Observations	2,835	2,835	2,835	2,835	2,835	2,835	2,835	2,835	2,835	2,835

	(11)	(12)	(13)	(14)	(15)	(16)	(17)	(18)
	SDG10	SDG11	SDG12	SDG13	SDG14	SDG15	SDG16	SDG17
Low Income Countries								
ODA received (log)	-0.002	-0.147	0.035	-0.133	-0.262	-1.264***	-0.158	0.032
	(0.168)	(0.174)	(0.028)	(0.118)	(0.205)	(0.354)	(0.112)	(0.135)
ODA received (log) * Low Income Countries	-0.444	-0.594	0.007	0.030	0.176	0.183	-1.104**	-0.012
	(0.339)	(0.751)	(0.072)	(0.268)	(0.436)	(0.332)	(0.525)	(0.517)
Sub-Saharan Africa (SSA)								
ODA received (log)	-0.037	-0.233	0.033*	-0.123	-0.290	-1.249***	-0.289*	-0.011
	(0.164)	(0.230)	(0.018)	(0.120)	(0.209)	(0.340)	(0.170)	(0.149)
ODA received (log) * SSA	-0.277	-0.078	-0.082	-0.124	0.564	0.185	-0.205	0.660
	(0.389)	(0.526)	(0.108)	(0.173)	(0.627)	(0.350)	(0.276)	(0.446)
Landlocked Developed Countries (LLDC)								
ODA received (log)	-0.034	-0.278	0.039**	-0.112	-0.220	-1.242***	-0.319*	0.017
	(0.163)	(0.225)	(0.019)	(0.118)	(0.220)	(0.340)	(0.169)	(0.142)
ODA received (log) *LLDC	-0.504	0.987	-0.269	-0.414	-0.887**	0.065	0.476	0.395
	(0.723)	(0.972)	(0.208)	(0.591)	(0.415)	(0.541)	(0.525)	(0.650)
English								
ODA received (log)	-0.086	-0.192	0.031	-0.121	-0.339	-1.317***	-0.326*	0.013
	(0.166)	(0.222)	(0.027)	(0.122)	(0.578)	(0.338)	(0.167)	(0.144)
ODA received (log) * English	0.543	-0.761	0.085**	-0.175	1.696**	1.319***	0.492	0.280
	(0.368)	(0.630)	(0.036)	(0.221)	(0.790)	(0.488)	(0.414)	(0.601)
Christian								
ODA received (log)	-0.048	0.103	0.006	-0.031	-0.242	-0.555**	-0.345	-0.100
	(0.159)	(0.286)	(0.038)	(0.153)	(0.213)	(0.253)	(0.216)	(0.194)
ODA received (log) * Christian	-0.008	-0.521**	0.043	-0.143	-0.009	-1.045***	0.062	0.190
	(0.163)	(0.241)	(0.030)	(0.106)	(0.176)	(0.282)	(0.123)	(0.157)
Islam								
ODA received (log)	-0.093	-0.359*	0.035	-0.221**	-0.341	-1.441***	-0.225*	0.170
	(0.182)	(0.200)	(0.030)	(0.106)	(0.339)	(0.358)	(0.122)	(0.122)
ODA received (log) * Islam	0.170	0.539	0.003	0.453*	0.353	0.981***	-0.361	-0.656**
	(0.267)	(0.544)	(0.056)	(0.245)	(0.897)	(0.337)	(0.372)	(0.312)
Colonial history								
ODA received (log)	-0.146	-0.235	0.018	-0.204	-0.294	-1.174***	-0.214*	-0.015
	(0.208)	(0.199)	(0.014)	(0.125)	(0.244)	(0.414)	(0.127)	(0.147)
ODA received (log) * Colony	0.211	-0.005	0.027	0.173	0.071	-0.161	-0.203	0.105
	(0.221)	(0.266)	(0.032)	(0.146)	(0.262)	(0.428)	(0.208)	(0.185)
Country FE	Yes	Yes	Yes	Yes	Yes	Yes	Yes	Yes
Year FE	Yes	Yes	Yes	Yes	Yes	Yes	Yes	Yes
Observations	2,835	2,835	2,835	2,835	2,835	2,835	2,835	2,835

Standard errors in parentheses. * $p<0.10$, ** $p<0.05$, *** $p<0.01$. We only keep the main effect for the ODA and interaction term of ODA to save the space. We excluded 23 countries that do not receive ODA from this sample and five countries due to data availability (Venezuela, Somalia, South Sudan, Djibouti, and Serbia). This also explains the different observations compared to the summary statistic. The control variable are consistent with Table 4

## Discussions and implications

The UN’s 2030 agenda outlines 17 SDGs to promote human and environmental development. ODA is key in financing these goals for developing countries. According to the UN, to achieve the SDGs, close to US$ 3.3-4.5 trillion per year needs to be mobilized, with the lion’s share of the financing gap facing developing economies (UN Sustainable Development Group 2024). However, existing research on SDGs and ODA neglects spatial factors, which may lead to biased policy evaluations. This study aims to fill this gap by examining two critical questions: (1) is there spatial autocorrelation in SDGs performance among countries, and (2) whether ODA contribute statistically significantly to achieving SDGs and what is the effect size of this relationship?

The results of Moran’s I analysis confirm spatial autocorrelation in the SDG index, suggesting that SDG achievement is not randomly distributed but shaped by shared socioeconomic and environmental conditions among neighboring countries. The spatial model further reveals that the impact of ODA on SDG performance is highly heterogeneous. ODA shows statistically significant positive effects on SDG 1 (no poverty), SDG 2 (zero hunger), and SDG 6 (clean water and sanitation), indicating its alignment with basic needs. However, no positive association is found for most other goals, and statistically significant negative effects emerge for SDG 8 (decent work and economic growth), SDG 9 (industry, innovation and infrastructure), SDG 15 (life on land), and SDG 16 (peace, justice and strong institutions). These adverse outcomes are consistent with research highlighting macroeconomic distortions (e.g., Dutch disease), weakened institutional accountability, and environmental degradation as unintended consequences of large aid inflows. Moreover, the variation in ODA effectiveness across country groups suggests that SDG implementation may follow different priorities depending on local context. Overall, the results imply that while ODA supports foundational human development, it may hinder progress in areas such as industrialization, governance, and environmental sustainability, echoing previous findings that donor assistance often favors immediate social needs over long-term structural goals^42^.

One possible reason why ODA has limited effectiveness in promoting the achievement of SDGs may be attributed to the small total amount of ODA. One study^97^ proposes an analytical framework for assessing the investment needs required to achieve the SDGs by 2030. The framework translates the 17 SDGs into eight investment areas and integrates the investment needs for climate change adaptation and mitigation with the development needs for each area. One study^97^ estimates that incremental spending needs in low- and lower-middle-income countries may amount to at least US$ 2013.1 trillion per year, with approximately half of these investments in the SDGs privately financed. Domestic resource mobilization can increase considerably, but an external financing gap of US$ 152-163 billion per year must be met through international public finance, including ODA. ODA is recognized as an important resource but is deemed inadequate to meet the trillions estimated to be required to achieve the SDGs^26^. Before the SDG, the MDGs were also primarily financed through ODA or foreign aid, with donor meetings encouraging traditional donors to meet their commitment to provide 0.7% of gross national income in ODA. However, few donors have ever met this target, and it is unlikely that they will under current definitions^26^. The 2030 Agenda reaffirms the importance of development cooperation and ODA, reinforcing the target for providers to allocate at least 0.7% of their gross national income in ODA to support LDCs and vulnerable contexts while mobilizing additional financial resources^98^. Recent evidence further underscores these challenges, One study^99^ warns that the sudden withdrawal of nearly half of global nutrition funding, along with dismantling USAID and statistically significant aid cuts by major Western donors, risks reversing decades of progress, especially undermining SDG 1 (no poverty) and SDG 2 (zero hunger).

Specifically, another study^100^ discusses the trends and issues related to ODA in Africa, with a focus on scholarships and education. The authors analyze ODA flows for the period of 2006 to 2015 and establish a baseline composite index based on data from the UNESCO Institute for Statistics and the SDGs 2018 tier indicators for SDG 4 (quality education). The findings reveal crucial gaps in the volume of ODA for scholarships, and the authors recommend increasing ODA for scholarships as well as the net enrollment rate and the percentage of trained primary teachers. The authors also suggest additional domestic funding and data collection to populate missing data on indicators, including the percentage of trained secondary school teachers.

In addition to the limited amount of ODA, the allocation of ODA was fragmented and increasingly dispersed, and some types of targets received more ODA than others. One study^101^ analyzes ODA spending on SDG 8.7 (end modern slavery, trafficking, and child labour). The study finds that ODA spending on this target is fragmented and dispersed, with only a few countries receiving the bulk of commitments. Certain types of exploitation received more ODA than others, with the largest pool of ODA commitments targeting human trafficking in recent years. However, ODA spending addressing other aspects of Target 8.7 was generally low^101^.

To achieve the SDGs, there is a need to shift from mobilizing billions to trillions of financial resources, which implies a requirement for new collaborations, including public-private partnerships (PPPs), since overseas development assistance alone is inadequate^102^. One study^27^ focuses on the fiscal challenges of 59 Low-Income Developing Countries (LIDCs) to achieve the SDGs. They argued that closing the budget gap to achieve SDGs in LIDCs can be done through increased domestic revenues, ODA, and Private Development Assistance (PDA). However, increased domestic revenues alone will not be sufficient. To reach the necessary SDG-directed development aid of US$ 300-US$ 400 billion per year, both ODA and PDA need to be increased^27,103^. Implementing a long-standing target of 0.7% of donor GNI allocated to ODA directed to SDGs would reduce the SDG funding gap in LIDCs by more than half. Additional funding of around US$ 100 billion can be mobilized through a one percent SDG wealth tax or voluntary philanthropy from the world’s billionaires. The logic runs that given the staggering amounts required to meet the investment gap, especially in poorer countries, ODA and other public finance forms should facilitate larger private finance flows for development. The idea is to use blended finance and financial instruments like debt and equity finance for PPPs to unlock, catalyze, and leverage private finance. Donors are now promoting de-risking investment as one of their roles through guarantees and finance deals. The shift from ODA to the wider category of development finance is not just about the SDGs, but the SDG framework provides a common narrative and institutional interfaces for strengthening state-private capital collaborations^26^.

Several economic, institutional, and political factors also affect the attraction and effective utilization of ODA. One study^25^ involves a literature review and an analysis of financial flow data in Africa to identify the main SDGs targeted by ODA. The results show a statistically significant disparity between academic research priorities related to SDGs and actual financial flows for SDGs in Africa, which could lead to the inefficient allocation of resources across sustainability domains. The authors suggest five categories of solutions, including capacity building, liberalization and deregulation, regulation and incentives, partnerships, and regional integration, to overcome the obstacles to attracting FDIs and ODA in Africa. However, another study^104^ finds that SDG partnerships are unequally distributed, with Northern and higher-income countries far more involved than their Southern and low-income counterparts. Such disparities risk reinforcing global inequalities, highlighting the need for more inclusive collaboration to achieve the 2030 Agenda. Additionally, official and private development assistance will be effective only if the funds are used responsibly. One study^27^ argues that it is feasible to effectively deploy funds by using pooled financing mechanisms like the Global Fund to Fight AIDS, TB, and Malaria (GFATM) and the Global Alliance on Vaccines and Immunizations (GAVI). These mechanisms have shown how to pool donor funds efficiently and manage them with professionalism and oversight over the past 15 years^97,105^.

The effective regulation of ODA funds being utilized towards the various SDGs necessitates the precise recording of each ODA transaction and its allocation towards a specific SDG, which remains an exceedingly challenging task. Due to the large number of transactions submitted to OECD Development Co-operation Directorate (DCD) each year, it would be time-consuming to manually attribute SDGs to each project. One study^94^ presented the capacity for supervised machine learning techniques in dealing with this challenge, such as text mining. Using text descriptions of more than 250,000 projects reported to the Creditor Reporting System (CRS)^1^ as input for categorization, the authors^94^ attribute the ODA recorded in CRS to each SDG. The new methodology explores the potential for today’s more powerful computing capacity to identify the relations between CRS text descriptions and their contribution to the SDGs.

## Conclusions

This study emphasizes the importance of a comprehensive approach to ODA that takes into account the spatial autocorrelation and heterogeneity of SDG performance and diverse needs, as tracked by the SDR2022. The results highlight the limited scope of ODA, as it mainly focuses on SDGs, focusing on the satisfaction of the very basic but most severe human needs (SDG 1 (no poverty) and SDG 2 (zero hunger). However, it shows no positive impact on most other goals, and even exhibits statistically significant negative associations with SDG 8 (decent work and economic growth), SDG 9 (industry, innovation and infrastructure), SDG 15 (life on land), and SDG 16 (peace, justice and strong institutions). These results point to two key implications: first, the scope of ODA should be broadened to support underfunded areas such as gender equality, environmental protection, and institutional capacity; second, aid strategies should be tailored to national contexts by recognizing trade-offs and complementarities among SDGs, enabling countries to prioritize and sequence goals that align with their development needs and implementation capacity.

The findings of this study are relevant for policymakers and have important policy implications for donor countries and international organizations providing ODA to developing countries. Firstly, donors should consider expanding the scope of their assistance to cover a broader range of SDGs, beyond just basic needs. This could include support for initiatives related to gender equality, environmental protection, and other SDGs that are not currently prioritized. Secondly, it is important for donors to recognize the heterogeneity in the effects of ODA on SDG performance and prioritize their assistance accordingly, more diverse strategies for different SDGs need to be provided. For example, if a particular country is lagging behind in achieving certain SDGs, donor countries should consider providing more assistance in those areas to help bridge the gap. Thirdly, international organizations providing ODA should work closely with developing countries to identify their specific needs and priorities in achieving SDGs. This could involve conducting needs assessments and developing customized assistance programs that address the unique challenges facing each country.

Future research could be based on a more granular analysis of sector-specific aid effectiveness to identify the most effective interventions and the conditions under which ODA translates into meaningful progress across diverse SDGs. Additionally, investigating the role of governance, institutional capacity, and local implementation strategies in mediating the impact of ODA could provide valuable insights into optimizing aid distribution. Future studies could also employ spatial econometric models to better understand the geographic spillover effects of ODA, ensuring that assistance is not only effectively targeted but also generates broader regional benefits.

## Supplementary Information

Supplementary Information.

## Data Availability

The datasets used and analysed during the current study available from the corresponding author on reasonable request.

## References

[1] Dlouhá, Jana & Pospíšilová, Marie. Education for sustainable development goals in public debate: The importance of participatory research in reflecting and supporting the consultation process in developing a vision for czech education. *J. Cleaner Prod.* **172**, 4314–4327 (2018).

[2] Sachs, Jeffrey D. et al. Six transformations to achieve the sustainable development goals. *Nature Sustain.* **2**(9), 805–814 (2019).

[3] Barbier, Edward B. & Burgess, Joanne C. Sustainable development goal indicators: Analyzing trade-offs and complementarities. *World Dev.* **122**, 295–305 (2019).

[4] Jacob, Arun. Mind the gap: Analyzing the impact of data gap in millennium development goals’(mdgs) indicators on the progress toward mdgs. *World Dev.* **93**, 260–278 (2017).

[5] Jeffrey Sachs, Guido Schmidt-Traub, Christian Kroll, Guillaume Lafortune, Grayson Fuller, and Finn Woelm. The sustainable development goals and covid-19. *Sustainable Development Report*, 2020, 2020.

[6] Jeffrey Sachs, Christian Kroll, Guillaume Lafortune, Grayson Fuller, and Finn Woelm. The decade of action for the sustainable development goals: Sustainable development report 2021. *Published online at sdgindex. org, Cambridge, UK Retrieved from:* https://unstatsun.org/sdgs/report/2020/(Accessed 5/11/2020), 2021.

[7] Jeffrey D. Sachs, Christian Kroll, Guillame Lafortune, Grayson Fuller, and Finn Woelm. *Sustainable development Report 2022*. Cambridge University Press, 2022.

[8] Moinuddin, Mustafa & Olsen, Simon Høiberg. Examining the unsustainable relationship between sdg performance, ecological footprint and international spillovers. *Sci. Rep.* **14**(1), 11277 (2024). 38760430 10.1038/s41598-024-61530-4 PMC11101620

[9] Annan-Diab, Fatima & Molinari, Carolina. Interdisciplinarity: Practical approach to advancing education for sustainability and for the sustainable development goals. *Int. J. Manag. Educ.* **15**(2), 73–83 (2017).

[10] Biggeri, Mario, Clark, David A., Ferrannini, Andrea & Mauro, Vincenzo. Tracking the sdgs in an integrated manner: A proposal for a new index to capture synergies and trade-offs between and within goals. *World Dev.* **122**, 628–647 (2019).

[11] Chimhowu, Admos O., Hulme, David & Munro, Lauchlan T. The ‘new’national development planning and global development goals: Processes and partnerships. *World Dev.* **120**, 76–89 (2019).

[12] Galli, Alessandro, Djurovic, Gordana, Hanscom, Laurel & Knežević, Jelena. Think globally, act locally: Implementing the sustainable development goals in montenegro. *Environ. Sci. Policy* **84**, 159–169 (2018).

[13] Hák, Tomáš, Janoušková, Svatava & Moldan, Bedřich. Sustainable development goals: A need for relevant indicators. *Ecol. Ind.* **60**, 565–573 (2016).

[14] Orenstein, Daniel E. & Shach-Pinsley, Dalit. A comparative framework for assessing sustainability initiatives at the regional scale.. *World Dev.* **98**, 245–256 (2017).

[15] Toetzke, Malte, Banholzer, Nicolas & Feuerriegel, Stefan. Monitoring global development aid with machine learning. *Nature Sustain.* **5**(6), 533–541 (2022).

[16] Bebbington, Jan & Unerman, Jeffrey. *Achieving the united nations sustainable development goals: an enabling role for accounting research* (Auditing & Accountability Journal, Accounting, 2018).

[17] Fuso Nerini, Francesco et al. Connecting climate action with other sustainable development goals. *Nature Sustainability* **2**(8), 674–680 (2019).

[18] Campagnolo, Lorenza & Davide, Marinella. Can the paris deal boost sdgs achievement? an assessment of climate mitigation co-benefits or side-effects on poverty and inequality. *World Dev.* **122**, 96–109 (2019).

[19] Blesh, Jennifer, Hoey, Lesli, Jones, Andrew D., Friedmann, Harriet & Perfecto, Ivette. Development pathways toward zero hunger. *World Dev.* **118**, 1–14 (2019).

[20] Allen, Cameron, Metternicht, Graciela, Wiedmann, Thomas & Pedercini, Matteo. Greater gains for australia by tackling all sdgs but the last steps will be the most challenging. *Nature Sustain.* **2**(11), 1041–1050 (2019).

[21] Biggeri, Mario, Carraro, Alessandro, Ciani, Federico & Romano, Donato. Disentangling the impact of a multiple-component project on sdg dimensions: The case of durum wheat value chain development in oromia (ethiopia). *World Dev.* **153**, 105810 (2022).

[22] Horn, Philipp & Grugel, Jean. The sdgs in middle-income countries: Setting or serving domestic development agendas? evidence from ecuador. *World Dev.* **109**, 73–84 (2018).

[23] Dawes, J. H. P. Sdg interlinkage networks: Analysis, robustness, sensitivities, and hierarchies. *World Dev.* **149**, 105693 (2022).

[24] Trisos, Christopher H. et al. Mosquito net fishing exemplifies conflict among sustainable development goals. *Nature Sustainability* **2**(7), 5–7 (2019).

[25] Julia Lopes, Albert Novas Somanje, Esteban Velez, Rodolfo Dam Lam, and Osamu Saito. Determinants of foreign investment and international aid for meeting the sustainable development goals in africa: a visual cognitive review of the literature. *Sustainability Challenges in Sub-Saharan Africa I: Continental Perspectives and Insights from Western and Central Africa*, pages 161–187, 2020.

[26] Mawdsley, Emma. From billions to trillions’ financing the sdgs in a world ‘beyond aid. *Dialog. Human Geograph.* **8**(2), 191–195 (2018).

[27] Jeffrey Sachs, Ms Vanessa Fajans-Turner, Ms Taylor Smith, Ms Cara Kennedy-Cuomo, Ms Teresa Parejo, and Mr Siamak Sam Loni. Closing the sdg budget gap. 2018.

[28] The World Bank. Net oda received (% of gni), 2023a. Accessed: 2023-02-24.

[29] Mary, Sebastien, Shaw, Kelsey, Colen, Liesbeth & y Paloma, Sergio Gomez. Does agricultural aid reduce child stunting?. *World Dev.* **130**, 104951 (2020).

[30] Dreher, Axel, Nunnenkamp, Peter & Thiele, Rainer. Does us aid buy un general assembly votes? a disaggregated analysis. *Public Choice* **136**(1), 139–164 (2008).

[31] Cruzatti, John, Dreher, Axel & Matzat, Johannes. Chinese aid and health at the country and local level. *World Dev.* **167**, 106214 (2023).

[32] Dreher, Axel, Gehring, Kai & Klasen, Stephan. Gesture politics or real commitment? gender inequality and the allocation of aid. *World Dev.* **70**, 464–480 (2015).

[33] Khomba, Daniel Chris & Trew, Alex. Aid and local growth in malawi. *J. Dev. Stud.* **58**(8), 1478–1500 (2022).

[34] Nowak-Lehmann, Felicitas, Dreher, Axel, Herzer, Dierk, Klasen, Stephan & Martínez-Zarzoso, Inmaculada. Does foreign aid really raise per capita income? a time series perspective. *Canad. J. Econ. /Revue Canad. d’économique* **45**(1), 288–313 (2012).

[35] Yahyaoui, Ismahene & Bouchoucha, Najeh. The long-run relationship between oda, growth and governance: An application of fmols and dols approaches. *Af. Dev. Rev.* **33**(1), 38–54 (2021).

[36] Rajan, Raghuram G. & Subramanian, Arvind. Aid, dutch disease, and manufacturing growth. *J. Develop. Econ.* **94**(1), 106–118 (2011).

[37] Bare, Matthew, Kauffman, Craig & Miller, Daniel C. Assessing the impact of international conservation aid on deforestation in sub-saharan africa. *Environ. Res. Lett.* **10**(12), 125010 (2015).

[38] Bräutigam, Deborah A. & Knack, Stephen. Foreign aid, institutions, and governance in sub-saharan africa. *Econ. Develop. Cult. Change* **52**(2), 255–285 (2004).

[39] Nunn, Nathan & Qian, Nancy. Us food aid and civil conflict. *Am. Econ. Rev.* **104**(6), 1630–1666 (2014).

[40] Dreher, Axel, Klasen, Stephan, Vreeland, James Raymond & Werker, Eric. The costs of favoritism: is politically driven aid less effective?. *Econ. Dev. Cultural Change* **62**(1), 157–191 (2013).

[41] Iacobuţă, Gabriela Ileana, Brandi, Clara, Dzebo, Adis & Duron, Sofia Donaji Elizalde. Aligning climate and sustainable development finance through an sdg lens. the role of development assistance in implementing the paris agreement. *Global Environ. Change* **74**, 102509 (2022).

[42] Thiele, Rainer, Nunnenkamp, Peter & Dreher, Axel. Do donors target aid in line with the millennium development goals? a sector perspective of aid allocation. *Rev. World Econ.* **143**, 596–630 (2007).

[43] Jeffrey D. Sachs. Government, geography, and growth: The true drivers of economic development. *Foreign Affairs*, 91 (5): 142–150, 2012. ISSN 00157120. URL http://www.jstor.org/stable/41720868.

[44] Nunn, Nathan & Puga, Diego. Ruggedness: The blessing of bad geography in africa. *Rev. Econ. Stat.* **94**(1), 20–36 (2012).

[45] Jeffrey M Wooldridge. *Econometric analysis of cross section and panel data*. MIT press, 2010.

[46] Moran, Patrick AP. The interpretation of statistical maps. *J. Roy. Stat. Soc.: Ser. B (Methodol.)* **10**(2), 243–251 (1948).

[47] Anselin, Luc. *Spatial econometrics: methods and models* (Springer Science & Business Media, Berlin Heidelberg, 1988).

[48] Roger S Bivand, Edzer J Pebesma, Virgilio Gomez-Rubio, and Edzer Jan Pebesma. *Applied spatial data analysis with R*, volume 747248717. Springer, 2008.

[49] FAO. Gadm 3.6 – administrative boundaries (level 1), 2023. Accessed: 2023-02-24.

[50] Anselin, Luc. Local indicators of spatial association-lisa. *Geogr. Anal.* **27**(2), 93–115 (1995).

[51] Kelejian, Harry H. & Prucha, Ingmar R. The relative efficiencies of various predictors in spatial econometric models containing spatial lags. *Reg. Sci. Urban Econ.* **37**(3), 363–374 (2007).

[52] Rosenzweig, Mark R. Population growth and human capital investments: theory and evidence. *J. Polit. Econ.* **98**((5, Part 2)), S38–S70 (1990).

[53] Cropper, Maureen & Griffiths, Charles. The interaction of population growth and environmental quality. *Am. Econ. Rev.* **84**(2), 250–254 (1994).

[54] d’Agostino, Giorgio, Dunne, J Paul & Pieroni, Luca. Government spending, corruption and economic growth. *World Dev.* **84**, 190–205 (2016).

[55] Alesina, Alberto & Dollar, David. Who gives foreign aid to whom and why?. *J. Econ. Growth* **5**(1), 33–63 (2000).

[56] Esfahani, Hadi Salehi & Ramírez, María Teresa. Institutions, infrastructure, and economic growth. *J. Dev. Econ.* **70**(2), 443–477 (2003).

[57] David N Weil. Health and economic growth. In *Handbook of economic growth*, volume 2, pages 623–682. Elsevier, 2014.

[58] Ross, Michael L. What have we learned about the resource curse?. *Annu. Rev. Polit. Sci.* **18**(1), 239–259 (2015).

[59] Dreher, Axel, Fuchs, Andreas, Parks, Brad, Strange, Austin M. & Tierney, Michael J. Apples and dragon fruits: The determinants of aid and other forms of state financing from china to africa.. *Int. Stud. Quart.* **62**(1), 182–194 (2018).

[60] Ahlin, Christian & Pang, Jiaren. Are financial development and corruption control substitutes in promoting growth?. *J. Dev. Econ.* **86**(2), 414–433 (2008).

[61] Isaksson, Ann-Sofie. & Kotsadam, Andreas. Chinese aid and local corruption. *J. Public Econ.* **159**, 146–159 (2018).

[62] Egger, Peter H. & Lassmann, Andrea. The language effect in international trade: A meta-analysis. *Econ. Lett.* **116**(2), 221–224 (2012).

[63] Lohmann, Johannes. Do language barriers affect trade?. *Econ. Lett.* **110**(2), 159–162 (2011).

[64] Felbermayr, Gabriel J. & Toubal, Farid. Cultural proximity and trade.. *Eur. Econ. Rev.* **54**(2), 279–293 (2010).

[65] Fensore, Irene, Legge, Stefan & Schmid, Lukas. Ancestry and international trade. *J. Comp. Econ.* **50**(1), 33–51 (2022).

[66] Noland, Marcus. Religion and economic performance. *World Dev.* **33**(8), 1215–1232 (2005).

[67] Becker, Sascha O. & Woessmann, Ludger. Was weber wrong? a human capital theory of protestant economic history. *Q. J. Econ.* **124**(2), 531–596 (2009).

[68] Nunn, Nathan. The long-term effects of africa’s slave trades. *Q. J. Econ.* **123**(1), 139–176 (2008).

[69] Acemoglu, Daron, Johnson, Simon & Robinson, James A. American Economic Review. *The colonial origins of comparative development: An empirical investigation* **91**(5), 1369–1401 (2001).

[70] Easterly, William & Levine, Ross. Tropics, germs, and crops: how endowments influence economic development. *J. Monet. Econ.* **50**(1), 3–39 (2003).

[71] Federico Belotti, Gordon Hughes, and Andrea Piano Mortari. Xsmle-a command to estimate spatial panel models in stata. *CEIS, University of Rome Tor Vergat School of Economics, University of Edinburg*, 2013.

[72] Belotti, Federico, Hughes, Gordon & Mortari, Andrea Piano. Spatial panel-data models using stata. *Stand. Genomic Sci.* **17**(1), 139–180 (2017).

[73] Li, Hongfei, Calder, Catherine A. & Cressie, Noel. Beyond moran’s i: testing for spatial dependence based on the spatial autoregressive model. *Geogr. Anal.* **39**(4), 357–375 (2007).

[74] Rüttenauer, Tobias. Spatial regression models: a systematic comparison of different model specifications using monte carlo experiments. *Sociological Methods & Research* **51**(2), 728–759 (2022).

[75] Mur, Jesús & Angulo, Ana. The spatial durbin model and the common factor tests. *Spat. Econ. Anal.* **1**(2), 207–226 (2006).

[76] Angrist, Joshua D. & Lavy, Victor. Using maimonides’ rule to estimate the effect of class size on scholastic achievement. *Q. J. Econ.* **114**(2), 533–575 (1999).

[77] Schmidt-Traub, Guido, Kroll, Christian, Teksoz, Katerina, Durand-Delacre, David & Sachs, Jeffrey D. National baselines for the sustainable development goals assessed in the sdg index and dashboards. *Nat. Geosci.* **10**(8), 547–555 (2017).

[78] Eleni Papadimitriou, Ana Rita Neves, and William Becker. *JRC Statistical Audit of the Sustainable Development Goals Index and Dashboards*. EUR 29776 EN, 2019.

[79] OECD. Net oda, 2023. Accessed: 2023-02-24.

[80] The World Bank. World development indicators, 2023b. Accessed: 2023-02-24.

[81] The Minerals UK. World mineral production, 2023. Accessed: 2023-02-24.

[82] Daron Acemoglu and James A Robinson. *Why nations fail: The origins of power, prosperity, and poverty*. Currency, 2012.

[83] Erik Voeten, Anton Strezhnev, and Michael Bailey. United nations general assembly voting data, 2009. URL 10.7910/DVN/LEJUQZ.

[84] Bailey, Michael A., Strezhnev, Anton & Voeten, Erik. Estimating dynamic state preferences from united nations voting data. *J. Conflict Resolut.* **61**(2), 430–456 (2017).

[85] Kaufmann, Daniel, Kraay, Aart & Mastruzzi, Massimo. The worldwide governance indicators: Methodology and analytical issues1. *Hague Journal on the Rule of Law* **3**(2), 220–246 (2011).

[86] The Worldwide Governance Indicators. Worldwide governance indicators, 2023. Accessed: 2023-01-24.

[87] The University of Groningen. World languages, 2016. Accessed: 2023-01-24.

[88] Maoz, Zeev & Henderson, Errol A. The world religion dataset, 1945–2010: Logic, estimates, and trends. *International Interactions* **39**(3), 265–291 (2013).

[89] UN. United nations office of the high representative for the least developed countries, landlocked developing countries and small island developing states search form, 2023a. Accessed: 2023-01-24.

[90] Nicholas Cox. Cipolate: Stata module for cubic interpolation. 2005.

[91] UN. Un country boundaries of the world, 2023b. Accessed: 2023-01-24.

[92] P Legendre and L Legendre. Numerical ecology. 3rd english ed. development in environmental modelling vol. 24, 2012.

[93] Andrew David Cliff and J Keith Ord. *Spatial processes: models & applications*. Taylor & Francis, 1981.

[94] Arnaud Pincet, Shu Okabe, and Martin Pawelczyk. Linking aid to the sustainable development goals: a machine learning approach. *OECD Development Co-operation Working Papers, No , OECD Publishing, Paris.*, 2019.

[95] Djeneba Doumbia and Morten Lykke Lauridsen. Closing the sdg financing gap: Trends and data. *EMCompass*, (73), 2019.

[96] United Nations Department of Economic and Social Affairs. The sustainable development goals report 2025. Technical report, United Nations, New York, 2025. Revision August 2025.

[97] Guido Schmidt-Traub and Jeffrey D Sachs. Financing sustainable development: implementing the sdgs through effective investment. *Sustainable Development Solution Network. Retrieved from:* https://irp-cdnmultiscreensite.com/be6d1d56/files/uploaded/150619-SDSN-Financing-Sustainable-Development-Paper-FINAL-02.pdf

[98] Alexandra Rudolph. *The concept of SDG-sensitive development cooperation: Implications for OECD-DAC members*. Number 1/2017. Discussion Paper, 2017.

[99] Osendarp, Saskia, Ruel, Marie, Udomkesmalee, Emorn, Tessema, Masresha & Haddad, Lawrence. The full lethal impact of massive cuts to international food aid. *Nature* **640**, 35–37 (2025). 40140717 10.1038/d41586-025-00898-3

[100] Godwell Nhamo, Charles Nhemachena, and Senia Nhamo. Emerging african picture of official development assistance and education-related sdgs indicators. *Scaling up SDGs Implementation: Emerging Cases from State, Development and Private Sectors*, pages 23–37, 2020.

[101] Kelly A Gleason and James Cockayne. Official development assistance and sdg target 8.7. 2018.

[102] Susan Horton. Financing the sustainable development goals: Beyond official development assistance. In *Achieving the Sustainable Development Goals*, pages 206–225. Routledge, 2019.

[103] Guido Schmidt-Traub and A Shah. Investment needs to achieve the sustainable development goals. *Paris and New York: Sustainable Development Solutions Network*, 2015.

[104] Blicharska, Malgorzata, Teutschbein, Claudia & Smithers, Richard J. Sdg partnerships may perpetuate the global north–south divide. *Sci. Rep.* **11**(1), 22092 (2021). 34824306 10.1038/s41598-021-01534-6 PMC8617181

[105] Sachs, Jeffrey D. & Schmidt-Traub, Guido. Global fund lessons for sustainable development goals. *Science* **356**(6333), 32–33 (2017). 28385974 10.1126/science.aai9380

