# Supplementary material for: A spatial perspective on the impact of official development assistance on sustainable development goals
Source: Sci Rep. 2026 Feb 1;16:5270. doi: 10.1038/s41598-026-35544-z (PMC12881366; doi:10.1038/s41598-026-35544-z)
Supplement: Supplementary file 1 — Supplementary Information. [file 41598_2026_35544_MOESM1_ESM.pdf]

References

OECD (2023). Net oda. Accessed: 2023-02-24.

Papadimitriou, E., Neves, A. R., & Becker, W. (2019). *JRC Statistical Audit of the Sustainable Development Goals Index and Dashboards*. EUR 29776 EN.

Pincet, A., Okabe, S., & Pawelczyk, M. (2019). Linking aid to the sustainable development goals: a machine learning approach. *OECD Development Co-operation Working Papers, No , OECD Publishing, Paris.*, .

Sachs, J. D., Kroll, C., Lafortune, G., Fuller, G., & Woelm, F. (2022). *Sustainable development Report 2022*. Cambridge University Press.

1 Supplementary Material

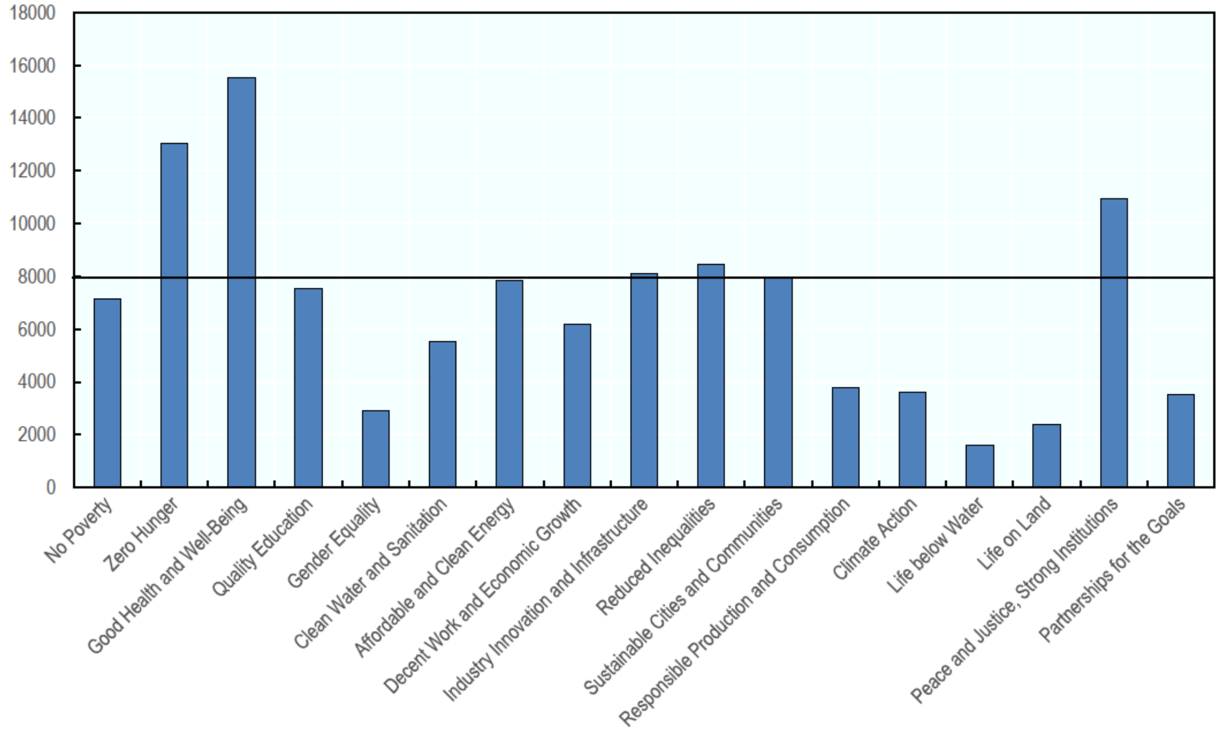

Figure A1: Aid per SDG for all DAC donors (US$ millions).
Source: Pincet et al. (2019).
Note: The red line is the mean value.

Table A1: 163 countries with SDG scores included in Sustainable Development Report.

ID	ISO3	Name
1	AFG	Afghanistan
2	AGO	Angola
3	ALB	Albania
4	ARE	United Arab Emirates
5	ARG	Argentina
6	ARM	Armenia
7	AUS	Australia
8	AUT	Austria
9	AZE	Azerbaijan
10	BDI	Burundi
11	BEL	Belgium
12	BEN	Benin
13	BFA	Burkina Faso
14	BGD	Bangladesh
15	BGR	Bulgaria
16	BHR	Bahrain
17	BIH	Bosnia & Herzegovina
18	BLR	Belarus
19	BLZ	Belize
20	BOL	Bolivia
21	BRA	Brazil
22	BRB	Barbados
23	BRN	Brunei Darussalam
24	BTN	Bhutan
25	BWA	Botswana
26	CAF	Central African Republic
27	CAN	Canada
28	CHE	Switzerland
29	CHL	Chile
30	CHN	China
31	CIV	Côte d'Ivoire
32	CMR	Cameroon
33	COD	Democratic Republic of the Congo
34	COG	Congo
35	COL	Colombia
36	CRI	Costa Rica
37	CUB	Cuba
38	CYP	Cyprus
39	CZE	Czech Republic
40	DEU	Germany
41	DJI	Djibouti
42	DNK	Denmark
43	DOM	Dominican Republic
44	DZA	Algeria
45	ECU	Ecuador
46	EGY	Egypt
47	ESP	Spain
48	EST	Estonia
49	ETH	Ethiopia
50	FIN	Finland
51	FJI	Fiji
52	FRA	France
53	GAB	Gabon
54	GBR	U.K. of Great Britain and Northern Ireland
55	GEO	Georgia
56	GHA	Ghana
57	GIN	Guinea
58	GMB	Gambia
59	GRC	Greece
60	GTM	Guatemala

Source: Sachs et al. (2022).

Table A1: 163 countries with SDG scores included in Sustainable Development Report – Continued.

ID	ISO3	Name
61	GUY	Guyana
62	HND	Honduras
63	HRV	Croatia
64	HTI	Haiti
65	HUN	Hungary
66	IDN	Indonesia
67	IND	India
68	IRL	Ireland
69	IRN	Iran (Islamic Republic of)
70	IRQ	Iraq
71	ISL	Iceland
72	ISR	Israel
73	ITA	Italy
74	JAM	Jamaica
75	JOR	Jordan
76	JPN	Japan
77	KAZ	Kazakhstan
78	KEN	Kenya
79	KGZ	Kyrgyzstan
80	KHM	Cambodia
81	KOR	Republic of Korea
82	KWT	Kuwait
83	LAO	Lao People's Democratic Republic
84	LBN	Lebanon
85	LBR	Liberia
86	LKA	Sri Lanka
87	LSO	Lesotho
88	LTU	Lithuania
89	LUX	Luxembourg
90	LVA	Latvia
91	MAR	Morocco
92	MDA	Moldova, Republic of
93	MDG	Madagascar
94	MDV	Maldives
95	MEX	Mexico
96	MKD	The former Yugoslav Republic of Macedonia
97	MLI	Mali
98	MLT	Malta
99	MMR	Myanmar
100	MNE	Montenegro
101	MNG	Mongolia
102	MOZ	Mozambique
103	MRT	Mauritania
104	MUS	Mauritius
105	MWI	Malawi
106	MYS	Malaysia
107	NAM	Namibia
108	NER	Niger
109	NGA	Nigeria
110	NIC	Nicaragua
111	NLD	Netherlands
112	NOR	Norway
113	NPL	Nepal
114	NZL	New Zealand
115	OMN	Oman
116	PAK	Pakistan
117	PAN	Panama
118	PER	Peru
119	PHL	Philippines
120	PNG	Papua New Guinea

Source: Sachs et al. (2022).

Table A1: 163 countries with SDG scores included in Sustainable Development Report – Continued.

ID	ISO3	Name
121	POL	Poland
122	PRT	Portugal
123	PRY	Paraguay
124	QAT	Qatar
125	ROU	Romania
126	RUS	Russian Federation
127	RWA	Rwanda
128	SAU	Saudi Arabia
129	SDN	Sudan
130	SEN	Senegal
131	SGP	Singapore
132	SLE	Sierra Leone
133	SLV	El Salvador
134	SOM	Somalia
135	SRB	Serbia
136	SSD	South Sudan
137	STP	Sao Tome and Principe
138	SUR	Suriname
139	SVK	Slovakia
140	SVN	Slovenia
141	SWE	Sweden
142	SWZ	Swaziland
143	SYR	Syrian Arab Republic
144	TCD	Chad
145	TGO	Togo
146	THA	Thailand
147	TJK	Tajikistan
148	TKM	Turkmenistan
149	TTO	Trinidad and Tobago
150	TUN	Tunisia
151	TUR	Turkey
152	TZA	United Republic of Tanzania
153	UGA	Uganda
154	UKR	Ukraine
155	URY	Uruguay
156	USA	United States of America
157	UZB	Uzbekistan
158	VEN	Venezuela
159	VNM	Vietnam
160	YEM	Yemen
161	ZAF	South Africa
162	ZMB	Zambia
163	ZWE	Zimbabwe

Source: Sachs et al. (2022).

Table A2: Conceptual framework of the SDG index.

SDG	Number of indicators
SDG1 No Poverty	2
SDG2 Zero Hunger	17
SDG3 Good Health and Well-being	14
SDG4 Quality Education	3
SDG5 Gender Equality	4
SDG6 Clean Water and Sanitation	5
SDG7 Affordable and Clean Energy	3
SDG8 Decent Work and Economic Growth	5
SDG9 Industry, Innovation and Infrastructure	6
SDG10 Reduced Inequality	1
SDG11 Sustainable Cities and Communities	3
SDG12 Responsible Consumption and Production	6
SDG13 Climate Action	4
SDG14 Life Below Water	4
SDG15 Life on Land	5
SDG16 Peace and Justice Strong Institutions	9
SDG17 Partnerships to Achieve the Goal	4

Source: Papadimitriou et al. (2019).

Table A3: 23 developed countries included in Sustainable Development Report but do not receive ODA.

ID	ISO3	Name
1	AUS	Australia
2	AUT	Austria
3	BEL	Belgium
4	CAN	Canada
5	CHE	Switzerland
6	DEU	Germany
7	DNK	Denmark
8	ESP	Spain
9	FIN	Finland
10	FRA	France
11	GBR	U.K. of Great Britain and Northern Ireland
12	GRC	Greece
13	IRL	Ireland
14	ISL	Iceland
15	ITA	Italy
16	JPN	Japan
17	LUX	Luxembourg
18	NLD	Netherlands
19	NOR	Norway
20	NZL	New Zealand
21	PRT	Portugal
22	SWE	Sweden
23	USA	United States of America

Source: Sachs et al. (2022) and OECD (2023).

Table A4: Average SDG index score from 2000 to 2022.

Year	SDG index score	SDG1	SDG2	SDG3	SDG4	SDG5	SDG6	SDG7	SDG8	SDG9
2000	60.84	69.99	56.33	57.81	64.42	48.58	62.81	58.21	64.52	19.51
2001	61.04	69.99	56.35	58.28	64.96	48.93	63.04	58.61	64.62	19.94
2002	61.28	69.99	56.40	58.71	65.55	49.33	63.24	59.28	64.51	20.58
2003	61.47	69.99	56.30	59.21	66.13	49.94	63.47	59.43	64.52	21.13
2004	61.76	69.99	56.70	59.75	66.82	50.52	63.67	59.76	64.67	21.70
2005	62.09	69.99	56.74	60.80	67.49	51.69	63.91	60.20	64.38	22.27
2006	62.37	69.99	56.98	61.23	68.31	52.07	64.16	60.66	64.79	22.92
2007	62.59	69.99	56.92	61.75	68.53	52.87	64.41	60.95	65.09	23.55
2008	62.81	69.99	57.24	62.38	68.90	53.17	64.63	61.20	65.22	24.40
2009	63.13	69.99	57.65	62.99	69.33	53.71	64.95	61.92	65.03	25.40
2010	63.54	69.99	57.76	64.78	70.13	54.98	65.18	62.35	64.85	26.60
2011	63.84	70.81	57.99	65.34	70.96	55.72	65.46	62.42	64.91	28.16
2012	64.26	71.43	58.02	65.78	71.66	56.64	65.69	63.08	64.91	30.74
2013	64.58	72.21	58.25	66.17	72.01	57.50	65.94	63.65	64.92	32.70
2014	65.10	72.70	58.79	66.52	73.11	58.27	66.21	63.83	66.12	34.76
2015	65.44	73.31	58.68	67.49	73.66	58.85	66.49	64.54	66.40	36.65
2016	65.74	73.66	58.66	67.86	73.99	59.44	66.73	65.03	66.50	37.58
2017	66.33	74.10	58.87	68.65	74.29	60.00	66.99	65.49	67.84	41.89
2018	66.74	74.65	59.27	69.12	75.27	60.43	67.25	65.75	68.02	43.36
2019	67.03	74.98	59.24	69.80	75.96	61.31	67.47	65.92	68.14	44.82
2020	67.04	73.99	59.24	69.57	76.02	61.53	67.65	65.92	67.20	45.70
2021	67.05	74.45	59.24	69.56	76.06	61.55	67.65	65.92	67.22	45.93
2022	67.17	75.35	59.24	69.56	76.32	61.57	67.65	65.92	67.48	46.17

Notes: the summary statistic include SDG score index for all countries (i.e., 163 countries) included in the Sustainable Development Report (Sachs et al., 2022).

Table A4: Average SDG index score from 2000 to 2022 – Continued.

Year	SDG10	SDG11	SDG12	SDG13	SDG14	SDG15	SDG16	SDG17
2000	58.47	67.63	84.94	80.21	60.64	60.46	65.17	54.52
2001	58.43	67.65	84.94	80.13	61.04	60.82	65.11	54.84
2002	58.66	67.66	84.94	80.10	61.35	61.27	65.08	55.15
2003	58.87	67.68	84.94	79.86	61.55	61.51	65.07	55.46
2004	58.75	67.70	84.94	79.81	61.83	62.63	65.14	55.47
2005	58.64	68.08	84.94	79.86	62.43	63.05	65.12	56.00
2006	59.20	68.10	84.94	79.71	62.50	63.49	65.17	56.12
2007	59.37	68.00	84.94	79.58	62.58	64.03	65.08	56.31
2008	59.72	67.84	84.94	79.55	62.70	64.37	65.06	56.47
2009	60.26	68.14	84.94	79.85	62.80	64.60	64.80	56.91
2010	60.54	68.54	84.94	79.68	62.81	64.87	65.29	56.87
2011	60.65	68.00	84.93	79.29	63.13	65.02	65.55	56.93
2012	60.58	68.35	84.79	79.55	63.48	65.12	65.59	56.99
2013	60.61	68.06	84.75	79.55	63.37	65.28	65.50	57.32
2014	60.57	69.42	84.59	79.74	63.70	65.35	65.58	57.44
2015	60.85	69.22	84.42	80.09	63.69	65.34	65.75	57.09
2016	61.07	70.45	84.32	80.62	63.70	65.40	65.56	57.08
2017	61.18	70.70	84.22	80.52	63.90	65.53	65.67	57.85
2018	61.35	70.62	84.13	80.30	64.16	65.57	66.40	58.90
2019	61.42	70.62	84.04	80.34	64.21	65.59	66.44	59.17
2020	61.42	70.40	84.04	80.86	64.21	65.78	66.78	59.33
2021	61.42	70.23	84.04	80.86	64.21	65.72	66.59	59.28
2022	61.68	70.27	84.04	80.26	64.76	65.72	66.59	59.28

Notes: the summary statistic include SDG score index for all countries included in the Sustainable Development Report (Sachs et al., 2022).

Table A5: Moran's I test for SDGs indices with spatial weights matrices.

	Neighbor (0-1)	Capitals distance	Nearest 3 capitals	Nearest 5 capitals
SDG index	0.733^{***}	0.077^{***}	0.726^{***}	0.716^{***}
SDG 1	0.794^{***}	0.079^{***}	0.670^{***}	0.679^{***}
SDG 2	0.329^{***}	0.014^{***}	0.375^{***}	0.384^{***}
SDG 3	0.688^{***}	0.075^{***}	0.722^{***}	0.723^{***}
SDG 4	0.651^{***}	0.054^{***}	0.632^{***}	0.611^{***}
SDG 5	0.548^{***}	0.025^{***}	0.575^{***}	0.545^{***}
SDG 6	0.708^{***}	0.068^{***}	0.637^{***}	0.650^{***}
SDG 7	0.419^{***}	0.042^{***}	0.498^{***}	0.481^{***}
SDG 8	0.480^{***}	0.039^{***}	0.500^{***}	0.479^{***}
SDG 9	0.529^{***}	0.050^{***}	0.614^{***}	0.614^{***}
SDG 10	0.671^{***}	0.114^{***}	0.697^{***}	0.713^{***}
SDG 11	0.573^{***}	0.047^{***}	0.589^{***}	0.567^{***}
SDG 12	0.543^{***}	0.041^{***}	0.645^{***}	0.634^{***}
SDG 13	0.356^{***}	0.023^{***}	0.499^{***}	0.511^{***}
SDG 14	0.159^{***}	0.005^{***}	0.142^{***}	0.140^{***}
SDG 15	0.350^{***}	0.045^{***}	0.516^{***}	0.440^{***}
SDG 16	0.482^{***}	0.054^{***}	0.586^{***}	0.555^{***}
SDG 17	0.316^{***}	0.021^{***}	0.354^{***}	0.309^{***}

Notes: This table reports Moran's I statistics based on various spatial weight matrices. "Neighbor (0-1)" represents a contiguity based weight matrix where $w_{ij} = 1$ if countries i and j share a geographical border, and $w_{ij} = 0$ otherwise. "Capitals distance" are distance-based weights calculated as as $w_{ij} = \exp\left[-\frac{1}{2}\left(\frac{d_{ij}}{D}\right)^2\right]$, where d_{ij} is the distance between the capital cities of countries i and j, and D is the average distance between all pairs of capital cities ($j \neq i$). "Nearest 3 capitals" and "Nearest 5 capitals" are K-nearest based weights define $w_{ij} = 1$ if country j is among the three or five shortest distances between the capital city of country i and the capital cities of all other countries, and $w_{ij} = 0$ otherwise. The choice of three or five neighbors reflects the average number of geographical neighbors for a country, approximately 3.5. These spatial weight matrices provide a comprehensive representation of spatial dependence for robust spatial analysis. * $p < 0.10$, ** $p < 0.05$, *** $p < 0.01$.

Table A6: Lagged ODA as an IV in the first-Stage estimation.

	ODA received (log)
Lag ODA received (log)	0.783***
	(0.010)
Population (log)	0.065
	(0.089)
GDP per capita (log)	-0.149**
	(0.063)
Petrolium (log)	-0.002
	(0.017)
UNGA vote index	0.042
	(0.035)
Corruption index	0.054
	(0.052)
R^2	0.692
Country FE	Yes
Year FE	Yes
Observations	2,835

Notes: Standard errors in parentheses. * $p < 0.10$, ** $p < 0.05$, *** $p < 0.01$. We excluded 23 countries that do not receive ODA from this sample and five countries due to data availability (Venezuela, Somalia, South Sudan, Djibouti, and Serbia). This also explains the different observations compared to the summary statistic.

Table A7: Moran I statistic and p-values for residuals.

	(1)<br>Moran I statistic	(2)<br>p-value
2022 SDG index score	-0.051	0.746
Goal 1 Score	0.033	0.257
Goal 2 Score	-0.028	0.620
Goal 3 Score	0.019	0.337
Goal 4 Score	-0.029	0.623
Goal 5 Score	-0.081	0.875
Goal 6 Score	-0.062	0.798
Goal 7 Score	-0.008	0.497
Goal 8 Score	0.060	0.142
Goal 9 Score	0.079	0.085
Goal 10 Score	0.014	0.361
Goal 11 Score	0.061	0.139
Goal 12 Score	0.056	0.157
Goal 13 Score	-0.052	0.752
Goal 14 Score	-0.017	0.551
Goal 15 Score	0.040	0.223
Goal 16 Score	-0.015	0.542
Goal 17 Score	-0.007	0.488
Observations	135	

Notes: The Moran's I is calculated based on basic binary geographical weighting matrix.

Table A8: Impact of ODA on SDG index scores with SAR model.

	(1) SDG index score	(2) SDG1	(3) SDG2	(4) SDG3	(5) SDG4	(6) SDG5	(7) SDG6	(8) SDG7	(9) SDG8	(10) SDG9
ODA received (log)	-0.161**	0.732***	0.252**	-0.049	0.247	0.086	0.402***	0.255	-0.346***	-2.083***
	(0.065)	(0.184)	(0.105)	(0.115)	(0.299)	(0.185)	(0.092)	(0.173)	(0.116)	(0.347)
Population (log)	2.315***	4.666*	4.907**	10.539***	17.199***	4.686	3.979**	4.608**	1.988	-5.108
	(0.738)	(2.784)	(2.036)	(1.910)	(5.777)	(3.062)	(1.913)	(2.328)	(1.406)	(7.464)
GDP per capita (log)	2.540***	12.007***	6.430***	4.981***	8.975***	-0.317	4.442***	1.983	2.602***	-0.339
	(0.688)	(2.324)	(1.273)	(1.457)	(2.981)	(1.739)	(1.151)	(1.813)	(0.825)	(2.650)
Petrolium (log)	0.001	-0.306	-0.075	-0.234	0.660	-0.032	-0.126	-0.025	0.103	0.712*
	(0.085)	(0.424)	(0.241)	(0.151)	(0.529)	(0.373)	(0.211)	(0.277)	(0.124)	(0.388)
UNGA vote index	-0.221	0.379	-0.063	-1.089**	-0.475	-1.252	0.374	0.410	-0.566*	0.933
	(0.204)	(0.895)	(0.377)	(0.489)	(1.109)	(0.889)	(0.490)	(0.564)	(0.322)	(0.895)
Corruption index	0.655**	0.646	0.080	1.801**	0.290	1.259	-0.343	0.621	0.578	2.199
	(0.291)	(1.019)	(0.658)	(0.705)	(1.491)	(0.965)	(0.469)	(1.202)	(0.489)	(1.408)
Spatial										
ρ	0.061**	0.047**	0.048***	0.105***	0.036*	0.014	0.026	0.021	0.031	0.085***
	(0.030)	(0.023)	(0.017)	(0.020)	(0.022)	(0.029)	(0.026)	(0.020)	(0.019)	(0.029)
R^2	0.223	0.461	0.167	0.000	0.017	0.011	0.134	0.008	0.159	0.056
Country FE	Yes	Yes	Yes	Yes	Yes	Yes	Yes	Yes	Yes	Yes
Year FE	Yes	Yes	Yes	Yes	Yes	Yes	Yes	Yes	Yes	Yes
Observations	2,835	2,835	2,835	2,835	2,835	2,835	2,835	2,835	2,835	2,835

Notes: Standard errors in parentheses. * $p < 0.10$, ** $p < 0.05$, *** $p < 0.01$. We excluded 23 countries that do not receive ODA from this sample and five countries due to data availability (Venezuela, Somalia, South Sudan, Djibouti, and Serbia). This also explains the different observations compared to the summary statistic.

Table A8: Impact of ODA on SDG index scores with SAR model – Continued.

	(11) SDG10	(12) SDG11	(13) SDG12	(14) SDG13	(15) SDG14	(16) SDG15	(17) SDG16	(18) SDG17
ODA received (log)	-0.073	-0.225	0.035	-0.138	-0.264	-1.268***	-0.314*	0.036
	(0.219)	(0.208)	(0.024)	(0.120)	(0.229)	(0.320)	(0.176)	(0.137)
Population (log)	-3.648	7.835***	1.333***	-0.517	-3.053	-6.320*	-1.738	-0.496
	(2.691)	(2.515)	(0.372)	(1.162)	(2.821)	(3.831)	(1.904)	(1.793)
GDP per capita (log)	-0.472	8.318***	-0.103	-3.855***	-4.279***	-1.515	2.526*	0.275
	(1.291)	(1.852)	(0.191)	(1.189)	(1.340)	(1.214)	(1.459)	(1.494)
Petrolium (log)	0.422**	-0.132	-0.100	-0.411	-0.242	-0.410**	0.174	-0.001
	(0.180)	(0.307)	(0.075)	(0.255)	(0.169)	(0.162)	(0.256)	(0.199)
UNGA vote index	-1.281*	-1.299	0.003	0.243	-0.006	0.129	-0.004	-0.519
	(0.657)	(0.838)	(0.052)	(0.254)	(0.411)	(0.465)	(0.475)	(0.623)
Corruption index	0.395	0.823	-0.123	-0.337	-0.467	1.226*	1.732***	0.780
	(0.938)	(0.885)	(0.084)	(0.551)	(0.817)	(0.665)	(0.586)	(0.752)
Spatial								
ρ	0.032**	0.058***	0.037**	0.045***	-0.021	0.013	0.053***	0.068***
	(0.015)	(0.014)	(0.015)	(0.012)	(0.015)	(0.021)	(0.016)	(0.012)
R^2	0.000	0.050	0.267	0.484	0.013	0.047	0.480	0.032
Country FE	Yes	Yes	Yes	Yes	Yes	Yes	Yes	Yes
Year FE	Yes	Yes	Yes	Yes	Yes	Yes	Yes	Yes
Observations	2835	2835	2835	2835	2835	2835	2835	2835

Notes: Standard errors in parentheses. * $p < 0.10$, ** $p < 0.05$, *** $p < 0.01$. We excluded 23 countries that do not receive ODA from this sample and five countries due to data availability (Venezuela, Somalia, South Sudan, Djibouti, and Serbia). This also explains the different observations compared to the summary statistic.

Table A9: Impact of ODA on SDG index scores with SEM model.

	(1) SDG index score	(2) SDG1	(3) SDG2	(4) SDG3	(5) SDG4	(6) SDG5	(7) SDG6	(8) SDG7	(9) SDG8	(10) SDG9
ODA received (log)	-0.170**	0.776***	0.271**	-0.004	0.270	0.056	0.445***	0.246	-0.351***	-2.138***
	(0.070)	(0.201)	(0.109)	(0.122)	(0.310)	(0.193)	(0.095)	(0.175)	(0.117)	(0.352)
Population (log)	2.461***	4.404	5.048**	10.855***	17.795***	4.184	4.249**	4.840**	2.095	-4.237
	(0.829)	(3.012)	(2.196)	(2.150)	(6.187)	(3.202)	(1.894)	(2.439)	(1.446)	(9.933)
GDP per capita (log)	2.642***	12.257***	6.667***	4.670***	9.186***	0.775	4.931***	2.020	2.592***	-0.305
	(0.660)	(2.697)	(1.281)	(1.402)	(3.030)	(1.756)	(1.223)	(1.807)	(0.838)	(2.914)
Petrolium (log)	-0.005	-0.293	-0.092	-0.207	0.648	-0.050	-0.174	-0.005	0.098	0.606
	(0.084)	(0.404)	(0.246)	(0.158)	(0.533)	(0.361)	(0.213)	(0.277)	(0.122)	(0.424)
UNGA vote index	-0.309	0.171	-0.195	-1.172**	-0.763	-1.294	0.364	0.328	-0.589*	0.924
	(0.212)	(0.882)	(0.392)	(0.510)	(1.150)	(0.919)	(0.479)	(0.578)	(0.322)	(0.926)
Corruption index	0.664**	0.586	0.128	1.750**	0.218	1.355	-0.586	0.724	0.575	2.279
	(0.285)	(1.044)	(0.660)	(0.747)	(1.524)	(0.967)	(0.479)	(1.205)	(0.485)	(1.416)
Spatial										
λ	0.066***	0.043*	0.040***	0.100***	0.032*	0.035**	-0.009	0.023*	0.015	0.096***
	(0.013)	(0.022)	(0.013)	(0.014)	(0.018)	(0.015)	(0.028)	(0.013)	(0.012)	(0.019)
R^2	0.242	0.497	0.204	0.012	0.025	0.000	0.146	0.010	0.165	0.090
Country FE	Yes	Yes	Yes	Yes	Yes	Yes	Yes	Yes	Yes	Yes
Year FE	Yes	Yes	Yes	Yes	Yes	Yes	Yes	Yes	Yes	Yes
Observations	2,835	2,835	2,835	2,835	2,835	2,835	2,835	2,835	2,835	2,835

Notes: Standard errors in parentheses. * $p < 0.10$, ** $p < 0.05$, *** $p < 0.01$. We excluded 23 countries that do not receive ODA from this sample and five countries due to data availability (Venezuela, Somalia, South Sudan, Djibouti, and Serbia). This also explains the different observations compared to the summary statistic.

Table A9: Impact of ODA on SDG index scores with SEM model – Continued.

	(11)<br>SDG10	(12)<br>SDG11	(13)<br>SDG12	(14)<br>SDG13	(15)<br>SDG14	(16)<br>SDG15	(17)<br>SDG16	(18)<br>SDG17
ODA received (log)	-0.069	-0.240	0.035	-0.159	-0.259	-1.256***	-0.319*	0.004
	(0.230)	(0.215)	(0.026)	(0.129)	(0.220)	(0.334)	(0.189)	(0.154)
Population (log)	-3.643	8.703***	1.320***	-0.211	-3.197	-5.839	-1.915	-0.675
	(2.831)	(2.800)	(0.413)	(1.282)	(2.769)	(3.941)	(2.126)	(1.991)
GDP per capita (log)	-0.365	8.686***	-0.183	-3.901***	-4.230***	-0.755	2.707*	-0.015
	(1.326)	(1.947)	(0.207)	(1.272)	(1.307)	(1.320)	(1.510)	(1.648)
Petrolium (log)	0.404**	-0.117	-0.094	-0.409	-0.245	-0.437***	0.161	-0.001
	(0.178)	(0.306)	(0.074)	(0.253)	(0.167)	(0.167)	(0.262)	(0.195)
UNGA vote index	-1.091	-1.343	0.003	0.320	-0.003	0.139	0.015	-0.467
	(0.785)	(0.834)	(0.053)	(0.274)	(0.425)	(0.464)	(0.488)	(0.645)
Corruption index	0.337	0.739	-0.117	-0.377	-0.492	1.202*	1.762***	0.862
	(0.937)	(0.891)	(0.086)	(0.559)	(0.814)	(0.677)	(0.593)	(0.766)
Spatial								
λ	0.024	0.064***	0.034**	0.042***	-0.025*	0.021	0.050***	0.066***
	(0.016)	(0.013)	(0.015)	(0.014)	(0.013)	(0.013)	(0.016)	(0.011)
R^2	0.000	0.057	0.285	0.536	0.011	0.051	0.487	0.016
Country FE	Yes	Yes	Yes	Yes	Yes	Yes	Yes	Yes
Year FE	Yes	Yes	Yes	Yes	Yes	Yes	Yes	Yes
Observations	2835	2835	2835	2835	2835	2835	2835	2835

Notes: Standard errors in parentheses. * $p < 0.10$, ** $p < 0.05$, *** $p < 0.01$. We excluded 23 countries that do not receive ODA from this sample and five countries due to data availability (Venezuela, Somalia, South Sudan, Djibouti, and Serbia). This also explains the different observations compared to the summary statistic.

Table A10: Impact of ODA on SDG index scores with SDM model.

	(1) SDG index score	(2) SDG1	(3) SDG2	(4) SDG3	(5) SDG4	(6) SDG5	(7) SDG6	(8) SDG7	(9) SDG8	(10) SDG9
ODA received (log)	-0.185**	0.716***	0.168	-0.068	0.095	0.025	0.351***	0.159	-0.314**	-2.008***
	(0.072)	(0.225)	(0.132)	(0.118)	(0.331)	(0.192)	(0.102)	(0.179)	(0.124)	(0.358)
Population (log)	2.262**	5.162	4.095*	10.120***	14.504**	2.086	4.094*	4.673*	1.318	-1.874
	(1.011)	(3.458)	(2.413)	(2.134)	(6.502)	(3.361)	(2.131)	(2.578)	(1.697)	(8.973)
GDP per capita (log)	2.463***	11.676***	6.416***	4.242***	9.625***	2.349	3.732***	2.321	2.261***	-0.150
	(0.639)	(2.196)	(1.279)	(1.364)	(2.955)	(1.717)	(1.124)	(1.915)	(0.818)	(2.809)
Petrolium (log)	-0.009	-0.352	-0.046	-0.164	0.526	-0.167	-0.130	-0.077	0.127	0.703*
	(0.091)	(0.402)	(0.234)	(0.145)	(0.525)	(0.313)	(0.179)	(0.285)	(0.121)	(0.388)
UNGA vote index	-0.237	0.330	-0.160	-0.909*	-1.005	-1.555*	0.471	0.230	-0.585*	0.921
	(0.246)	(0.889)	(0.403)	(0.511)	(1.149)	(0.896)	(0.446)	(0.589)	(0.325)	(0.950)
Corruption index	0.664**	0.586	0.084	1.772**	0.052	1.324	-0.446	0.831	0.514	2.257
	(0.295)	(1.024)	(0.637)	(0.736)	(1.467)	(0.935)	(0.478)	(1.191)	(0.480)	(1.386)
Spatially lagged co-variates										
ODA received (log)	0.028	0.078	0.068	0.098**	0.187*	0.009	0.109**	0.132**	-0.053	0.007
	(0.021)	(0.060)	(0.058)	(0.040)	(0.110)	(0.073)	(0.043)	(0.061)	(0.044)	(0.139)
Population (log)	0.035	-0.374	0.149	-0.299	0.804	0.954	-0.074	-0.405	0.605*	-1.470
	(0.461)	(0.757)	(0.453)	(0.565)	(1.018)	(0.652)	(0.503)	(0.611)	(0.325)	(1.716)
GDP per capita (log)	0.218	0.202	0.456	0.717**	-0.113	-1.016**	0.860***	0.186	0.011	0.155
	(0.489)	(0.370)	(0.311)	(0.281)	(0.664)	(0.402)	(0.268)	(0.315)	(0.169)	(0.925)
Petrolium (log)	-0.033	-0.114	0.113	0.066	-0.021	-0.077	-0.087	-0.147	-0.033	0.048
	(0.061)	(0.201)	(0.125)	(0.101)	(0.254)	(0.158)	(0.098)	(0.124)	(0.093)	(0.251)
UNGA vote Index	0.188	0.289	0.501**	0.482**	1.409***	0.245	0.142	0.621**	-0.172	0.256
	(0.117)	(0.369)	(0.217)	(0.227)	(0.507)	(0.314)	(0.207)	(0.287)	(0.163)	(0.390)
Corruption Index	0.025	0.581	-0.603**	0.137	1.046*	-0.028	-0.519*	-0.497	0.185	-0.181
	(0.233)	(0.514)	(0.246)	(0.300)	(0.569)	(0.385)	(0.285)	(0.363)	(0.192)	(0.597)
Spatial										
ρ	0.053	0.039*	0.035**	0.078**	0.024	0.022	-0.018	0.020	0.014	0.090***
	(0.140)	(0.021)	(0.016)	(0.031)	(0.020)	(0.025)	(0.029)	(0.016)	(0.018)	(0.034)
R^2	0.269	0.521	0.150	0.023	0.035	0.001	0.150	0.043	0.038	0.049
Country FE	Yes	Yes	Yes	Yes	Yes	Yes	Yes	Yes	Yes	Yes
Year FE	Yes	Yes	Yes	Yes	Yes	Yes	Yes	Yes	Yes	Yes
Observations	2,835	2,835	2,835	2,835	2,835	2,835	2,835	2,835	2,835	2,835

Notes: Standard errors in parentheses. * $p < 0.10$, ** $p < 0.05$, *** $p < 0.01$. We excluded 23 countries that do not receive ODA from this sample and five countries due to data availability (Venezuela, Somalia, South Sudan, Djibouti, and Serbia). This also explains the different observations compared to the summary statistic.

Table A10: Impact of ODA on SDG index scores with SDM model – Continued.

	(11) SDG10	(12) SDG11	(13) SDG12	(14) SDG13	(15) SDG14	(16) SDG15	(17) SDG16	(18) SDG17
ODA received (log)	0.004	-0.249	0.007	-0.159	-0.180	-1.169***	-0.233	-0.093
	(0.246)	(0.188)	(0.033)	(0.151)	(0.260)	(0.348)	(0.173)	(0.182)
Population (log)	-2.327	9.427***	0.963***	-0.315	-0.943	-5.098	-1.488	-0.390
	(2.798)	(3.159)	(0.321)	(1.536)	(3.037)	(4.524)	(2.464)	(2.020)
GDP per capita (log)	-1.035	7.934***	-0.272	-3.763***	-3.566**	0.670	2.078	-0.715
	(1.390)	(1.831)	(0.212)	(1.261)	(1.443)	(1.318)	(1.462)	(1.457)
Petrolium (log)	0.470**	-0.206	-0.094	-0.432*	-0.265	-0.512***	0.229	0.046
	(0.198)	(0.294)	(0.073)	(0.263)	(0.167)	(0.142)	(0.242)	(0.187)
UNGA vote index	-0.924	-1.146	0.024	0.292	0.001	-0.040	0.010	-0.274
	(0.787)	(0.805)	(0.059)	(0.293)	(0.435)	(0.495)	(0.490)	(0.658)
Corruption index	0.276	0.909	-0.112	-0.343	-0.343	1.210*	1.748***	0.876
	(0.946)	(0.893)	(0.082)	(0.563)	(0.801)	(0.670)	(0.604)	(0.760)
Spatially lagged co-variates								
ODA received (log)	-0.083	0.123**	0.018	0.027	-0.037	-0.039	-0.080	0.102
	(0.075)	(0.062)	(0.013)	(0.057)	(0.068)	(0.081)	(0.049)	(0.083)
Population (log)	-0.307	-1.053	0.125	-0.181	-0.920	-0.349	0.079	-0.229
	(0.664)	(0.908)	(0.078)	(0.435)	(0.609)	(0.837)	(0.502)	(0.432)
GDP per capita (log)	0.017	0.091	0.059*	-0.118	-0.193	-0.909**	0.290	0.634**
	(0.349)	(0.362)	(0.033)	(0.198)	(0.332)	(0.395)	(0.271)	(0.264)
Petrolium (log)	0.122	-0.249	-0.001	0.004	-0.029	-0.009	-0.067	0.078
	(0.158)	(0.188)	(0.021)	(0.086)	(0.120)	(0.121)	(0.143)	(0.118)
UNGA vote index	-0.512*	0.180	-0.036	-0.027	0.020	0.055	-0.070	-0.212
	(0.305)	(0.351)	(0.035)	(0.144)	(0.215)	(0.188)	(0.168)	(0.227)
Corruption index	0.582	0.417	-0.032	0.159	-0.346	0.127	-0.314	-0.389
	(0.458)	(0.382)	(0.039)	(0.211)	(0.383)	(0.355)	(0.271)	(0.329)
Spatial								
ρ	0.024	0.056***	0.039***	0.043***	-0.021	0.016	0.045***	0.059***
	(0.016)	(0.013)	(0.014)	(0.012)	(0.014)	(0.017)	(0.016)	(0.012)
R^2	0.005	0.112	0.255	0.412	0.007	0.016	0.506	0.050
Country FE	Yes	Yes	Yes	Yes	Yes	Yes	Yes	Yes
Year FE	Yes	Yes	Yes	Yes	Yes	Yes	Yes	Yes
Observations	2835	2835	2835	2835	2835	2835	2835	2835

Notes: Standard errors in parentheses. * $p < 0.10$, ** $p < 0.05$, *** $p < 0.01$. We excluded 23 countries that do not receive ODA from this sample and five countries due to data availability (Venezuela, Somalia, South Sudan, Djibouti, and Serbia). This also explains the different observations compared to the summary statistic.

Table A11: Impact of ODA on SDG index scores with SAC model: robustness check with less control variables.

	(1) SDG index score	(2) SDG1	(3) SDG2	(4) SDG3	(5) SDG4	(6) SDG5	(7) SDG6	(8) SDG7	(9) SDG8	(10) SDG9
ODA received (log)	-0.152**	0.536***	0.247**	-0.020	0.225	0.042	0.264***	0.308*	-0.307***	-2.047***
	(0.076)	(0.135)	(0.097)	(0.095)	(0.249)	(0.190)	(0.075)	(0.179)	(0.109)	(0.461)
Population (log)	2.245**	4.814**	4.528**	7.227***	14.810***	2.562	2.636**	5.535**	2.493**	-4.840
	(0.985)	(1.981)	(1.854)	(1.617)	(5.441)	(3.369)	(1.154)	(2.525)	(1.227)	(7.787)
GDP per capita (log)	2.868***	9.672***	5.823***	5.272***	8.161***	1.985	2.911***	2.196	2.502***	0.882
	(1.007)	(1.806)	(1.441)	(1.380)	(3.074)	(1.740)	(0.929)	(1.956)	(0.732)	(3.137)
Spatial										
ρ	0.062	0.124***	0.086**	0.142***	0.105***	-0.117**	0.131***	-0.019	0.076	0.089
	(0.087)	(0.025)	(0.038)	(0.020)	(0.037)	(0.057)	(0.023)	(0.109)	(0.059)	(0.072)
λ	0.001	-0.142***	-0.056	-0.098**	-0.106**	0.118***	-0.179***	0.036	-0.063	0.006
	(0.093)	(0.045)	(0.048)	(0.038)	(0.051)	(0.024)	(0.038)	(0.092)	(0.071)	(0.086)
R^2	0.241	0.141	0.110	0.001	0.004	0.029	0.037	0.004	0.074	0.056
Country FE	Yes	Yes	Yes	Yes	Yes	Yes	Yes	Yes	Yes	Yes
Year FE	Yes	Yes	Yes	Yes	Yes	Yes	Yes	Yes	Yes	Yes
Observations	2,835	2,835	2,835	2,835	2,835	2,835	2,835	2,835	2,835	2,835

Notes: Standard errors in parentheses. * $p < 0.10$, ** $p < 0.05$, *** $p < 0.01$. We excluded 23 countries that do not receive ODA from this sample and five countries due to data availability (Venezuela, Somalia, South Sudan, Djibouti, and Serbia). This also explains the different observations compared to the summary statistic.

Table A11: Impact of ODA on SDG index scores with SAC model: robustness check with less control variables – Continued.

	(11)<br>SDG10	(12)<br>SDG11	(13)<br>SDG12	(14)<br>SDG13	(15)<br>SDG14	(16)<br>SDG15	(17)<br>SDG16	(18)<br>SDG17
ODA received (log)	-0.036	-0.240	0.033	-0.136	-0.271	-1.255***	-0.269*	0.042
	(0.164)	(0.235)	(0.026)	(0.116)	(0.214)	(0.333)	(0.162)	(0.142)
Population (log)	-2.804	7.960*	1.225***	-0.976	-3.477	-6.778	-1.546	-0.585
	(2.114)	(4.187)	(0.440)	(1.287)	(2.610)	(4.161)	(1.727)	(1.836)
GDP per capita (log)	-0.104	8.987***	-0.350	-4.322***	-4.683***	-0.800	2.924*	0.769
	(1.011)	(2.286)	(0.239)	(1.113)	(1.146)	(1.282)	(1.577)	(1.603)
Spatial								
ρ	0.117***	0.027	-0.006	0.056***	0.022	-0.011	0.088***	0.065
	(0.029)	(0.191)	(.)	(0.021)	(0.085)	(0.038)	(0.028)	(0.053)
λ	-0.117**	0.038	0.048***	-0.019	-0.051	0.027	-0.060*	0.002
	(0.049)	(0.198)	(0.015)	(0.026)	(0.094)	(0.028)	(0.034)	(0.062)
R^2	0.002	0.076	0.322	0.412	0.010	0.047	0.436	0.055
Country FE	Yes	Yes	Yes	Yes	Yes	Yes	Yes	Yes
Year FE	Yes	Yes	Yes	Yes	Yes	Yes	Yes	Yes
Observations	2,835	2,835	2,835	2,835	2,835	2,835	2,835	2,835

Notes: Standard errors in parentheses. * $p < 0.10$, ** $p < 0.05$, *** $p < 0.01$. We excluded 23 countries that do not receive ODA from this sample and five countries due to data availability (Venezuela, Somalia, South Sudan, Djibouti, and Serbia). This also explains the different observations compared to the summary statistic.

Table A12: Impact of ODA on SDG index scores with SAC model: robustness check with core control variables.

	(1) SDG index score	(2) SDG1	(3) SDG2	(4) SDG3	(5) SDG4	(6) SDG5	(7) SDG6	(8) SDG7	(9) SDG8	(10) SDG9
ODA received (log)	-0.154*	0.570***	0.255***	0.015	0.256	0.039	0.287***	0.265	-0.310**	-2.009***
	(0.081)	(0.139)	(0.099)	(0.116)	(0.255)	(0.196)	(0.077)	(0.186)	(0.133)	(0.464)
Population (log)	0.870	-1.186	0.659	3.626***	7.878**	2.312	0.449	3.859	0.638	-4.792
	(0.782)	(1.613)	(1.266)	(1.330)	(3.666)	(3.391)	(0.926)	(2.565)	(1.392)	(7.150)
Spatial										
ρ	0.081***	0.157***	0.134***	0.163***	0.130***	-0.093	0.158***	-0.030	0.071	0.094
	(0.028)	(0.014)	(0.015)	(0.007)	(0.022)	(0.082)	(0.010)	(0.133)	(0.087)	(0.068)
λ	0.009	-0.157***	-0.105***	-0.099***	-0.126***	0.102**	-0.182***	0.052	-0.032	-0.007
	(.)	(0.038)	(0.026)	(0.025)	(0.036)	(0.044)	(0.031)	(0.107)	(0.104)	(0.085)
R^2	0.001	0.158	0.002	0.005	0.022	0.006	0.004	0.016	0.073	0.034
Country FE	Yes	Yes	Yes	Yes	Yes	Yes	Yes	Yes	Yes	Yes
Year FE	Yes	Yes	Yes	Yes	Yes	Yes	Yes	Yes	Yes	Yes
Observations	2,835	2,835	2,835	2,835	2,835	2,835	2,835	2,835	2,835	2,835

Notes: Standard errors in parentheses. * $p < 0.10$, ** $p < 0.05$, *** $p < 0.01$. We excluded 23 countries that do not receive ODA from this sample and five countries due to data availability (Venezuela, Somalia, South Sudan, Djibouti, and Serbia). This also explains the different observations compared to the summary statistic.

Table A12: Impact of ODA on SDG index scores with SAC model: robustness check with core control variables – Continued.

	(11) SDG10	(12) SDG11	(13) SDG12	(14) SDG13	(15) SDG14	(16) SDG15	(17) SDG16	(18) SDG17
ODA received (log)	-0.038	-0.297	0.022	-0.169	-0.237	-1.208***	-0.219	0.044
	(0.164)	(0.257)	(0.019)	(0.138)	(0.258)	(0.341)	(0.150)	(0.146)
Population (log)	-2.738	4.969	1.033**	1.542	0.019	-5.770	-2.751**	-1.008
	(1.971)	(3.499)	(0.438)	(1.392)	(2.853)	(3.854)	(1.269)	(1.951)
Spatial								
ρ	0.116***	-0.068	0.110***	0.062	-0.104**	-0.014	0.112***	0.063
	(0.029)	(0.051)	(0.032)	(0.066)	(0.044)	(0.044)	(0.016)	(0.055)
λ	-0.117**	0.123***	-0.111**	0.005	0.082**	0.037	-0.086***	0.007
	(0.048)	(0.030)	(0.055)	(0.076)	(0.033)	(0.033)	(0.029)	(0.061)
R^2	0.001	0.034	0.165	0.030	0.016	0.046	0.122	0.008
Country FE	Yes	Yes	Yes	Yes	Yes	Yes	Yes	Yes
Year FE	Yes	Yes	Yes	Yes	Yes	Yes	Yes	Yes
Observations	2,835	2,835	2,835	2,835	2,835	2,835	2,835	2,835

Notes: Standard errors in parentheses. * $p < 0.10$, ** $p < 0.05$, *** $p < 0.01$. We excluded 23 countries that do not receive ODA from this sample and five countries due to data availability (Venezuela, Somalia, South Sudan, Djibouti, and Serbia). This also explains the different observations compared to the summary statistic.

Table A13: Impact of ODA on SDG index scores with SAC model: robustness check with developed countries.

	(1) SDG index score	(2) SDG1	(3) SDG2	(4) SDG3	(5) SDG4	(6) SDG5	(7) SDG6	(8) SDG7	(9) SDG8	(10) SDG9
ODA received (log)	-0.179**	0.517***	0.193**	-0.069	0.123	0.030	0.232***	0.244	-0.317***	-1.976***
	(0.076)	(0.125)	(0.089)	(0.093)	(0.235)	(0.170)	(0.070)	(0.168)	(0.108)	(0.415)
Population (log)	1.969**	4.014**	4.893***	7.226***	13.265***	3.848	2.512***	4.713**	2.296**	-4.389
	(0.791)	(1.830)	(1.804)	(1.633)	(4.732)	(3.816)	(0.957)	(2.266)	(1.168)	(6.644)
GDP per capita (log)	2.200**	9.057***	6.113***	4.432***	7.308***	0.206	3.360***	2.330	2.253***	-0.776
	(0.860)	(1.600)	(1.357)	(1.343)	(2.595)	(2.781)	(0.773)	(1.790)	(0.732)	(2.312)
Petrolium (log)	0.033	-0.327	-0.038	-0.146	0.573	-0.007	-0.197	-0.040	0.073	0.766**
	(0.085)	(0.380)	(0.216)	(0.133)	(0.469)	(0.331)	(0.129)	(0.271)	(0.120)	(0.379)
UNGA vote index	-0.163	0.719	0.269	-0.565	0.630	-1.084	0.562*	0.395	-0.423	0.766
	(0.190)	(0.684)	(0.374)	(0.487)	(0.972)	(0.901)	(0.332)	(0.602)	(0.293)	(0.870)
Corruption index	0.580**	0.888	-0.018	1.582***	0.581	1.077	-0.550	0.537	0.552	1.955
	(0.284)	(0.821)	(0.599)	(0.582)	(1.206)	(0.946)	(0.364)	(1.138)	(0.438)	(1.351)
Spatial										
ρ	0.085	0.123***	0.093***	0.135***	0.105***	0.077	0.134***	-0.013	0.084**	0.097*
	(0.058)	(0.022)	(0.030)	(0.022)	(0.033)	(0.176)	(0.018)	(0.135)	(0.036)	(0.054)
λ	-0.026	-0.133***	-0.060	-0.086**	-0.109**	-0.046	-0.184***	0.036	-0.065	0.004
	(0.068)	(0.040)	(0.040)	(0.040)	(0.046)	(0.210)	(0.030)	(0.117)	(0.045)	(0.069)
R^2	0.364	0.240	0.200	0.022	0.032	0.001	0.111	0.040	0.212	0.131
Country FE	Yes	Yes	Yes	Yes	Yes	Yes	Yes	Yes	Yes	Yes
Year FE	Yes	Yes	Yes	Yes	Yes	Yes	Yes	Yes	Yes	Yes
Observations	3,318	3,318	3,318	3,318	3,318	3,318	3,318	3,318	3,318	3,318

Notes: Standard errors in parentheses. * $p < 0.10$, ** $p < 0.05$, *** $p < 0.01$. We excluded five countries due to the data availability (Venezuela, Somalia, South Sudan, Djibouti, and Serbia). This also explains the different observations compared to the summary statistic.

Table A13: Impact of ODA on SDG index scores with SAC model: robustness check with developed countries – Continued.

	(11) SDG10	(12) SDG11	(13) SDG12	(14) SDG13	(15) SDG14	(16) SDG15	(17) SDG16	(18) SDG17
ODA received (log)	-0.115	-0.202	0.038	-0.032	-0.238	-1.253***	-0.271*	0.054
	(0.166)	(0.211)	(0.027)	(0.118)	(0.209)	(0.325)	(0.163)	(0.138)
Population (log)	-1.935	8.112**	1.128***	-3.202**	-2.642	-6.006*	-2.305*	-0.389
	(2.052)	(3.279)	(0.378)	(1.343)	(2.142)	(3.575)	(1.394)	(1.799)
GDP per capita (log)	0.260	7.898***	-0.362*	-5.582***	-3.352***	-0.930	1.632	0.037
	(1.028)	(1.703)	(0.203)	(1.227)	(1.113)	(1.178)	(1.130)	(1.350)
Petrolium (log)	0.401**	-0.037	-0.080	-0.224	-0.245	-0.346**	0.190	-0.072
	(0.163)	(0.311)	(0.068)	(0.245)	(0.178)	(0.167)	(0.243)	(0.193)
UNGA vote index	-1.040	-1.328*	0.001	0.114	0.130	0.073	0.064	-0.504
	(0.657)	(0.779)	(0.053)	(0.262)	(0.412)	(0.448)	(0.443)	(0.583)
Corruption index	0.550	0.460	-0.096	-0.389	-0.610	1.076*	1.557***	1.175*
	(0.819)	(0.825)	(0.079)	(0.552)	(0.734)	(0.623)	(0.541)	(0.707)
Spatial	0.110***	-0.025	-0.017	0.073***	0.046	0.004	0.086***	0.069
	(0.030)	(0.092)	(.)	(0.015)	(0.049)	(0.046)	(0.024)	(0.043)
λ	-0.115**	0.088	0.055***	-0.020	-0.062	0.022	-0.039	-0.010
	(0.048)	(0.072)	(0.013)	(0.021)	(0.056)	(0.038)	(0.029)	(0.052)
R^2	0.000	0.145	0.285	0.304	0.011	0.044	0.512	0.081
Country FE	Yes	Yes	Yes	Yes	Yes	Yes	Yes	Yes
Year FE	Yes	Yes	Yes	Yes	Yes	Yes	Yes	Yes
Observations	3,318	3,318	3,318	3,318	3,318	3,318	3,318	3,318

Notes: Standard errors in parentheses. * $p < 0.10$, ** $p < 0.05$, *** $p < 0.01$. We excluded five countries due to the data availability (Venezuela, Somalia, South Sudan, Djibouti, and Serbia). This also explains the different observations compared to the summary statistic.
